# Contribution of protein–protein interactions to the endothelial-barrier-stabilizing function of KRIT1

**DOI:** 10.1242/jcs.258816

**Published:** 2022-01-25

**Authors:** Harsha Swamy, Angela J. Glading

**Affiliations:** Department of Pharmacology and Physiology, University of Rochester, Rochester, NY 14642, USA

**Keywords:** Adherens junction, Cavernous malformation, Vascular homeostasis

## Abstract

Krev-interaction trapped protein 1 (KRIT1) is an endothelial scaffold protein that promotes adherens junction (AJ) stability. The precise mechanism by which KRIT1 promotes barrier stabilization is unclear. We tested the ability of a panel of KRIT1 constructs containing mutations that inhibit Rap1 binding, ICAP1α binding, disrupt KRIT1's phosphotyrosine-binding (PTB) domain, or direct KRIT1 to the plasma membrane, either alone or in combination, to restore barrier function in KRIT1-deficient endothelial cells. We found that ablating the ^192^NPAY^195^ motif or disrupting the PTB domain was sufficient to restore AJ protein localization and barrier function to control levels, irrespective of the junctional localization of KRIT1 or Rap1 binding. The ability of our KRIT1 constructs to rescue AJ and barrier function in KRIT1-depleted endothelial cells correlated with decreased β1 integrin activity and maintenance of cortical actin fibers. Taken together, our findings indicate that Rap1 binding, ICAP1α binding and junctional localization are not required for the ability of KRIT1 to stabilize endothelial contacts, and suggest that the ability of KRIT1 to limit integrin activity could be involved in barrier stabilization.

## Abstract (precis)

**Summary:** Contrary to current models, KRIT1 does not require binding to ICAP1α and Rap1, or junctional localization, to stabilize the endothelial barrier. Instead, self-interaction and/or β1 integrin activation are critical elements of KRIT1 signaling.

## INTRODUCTION

Krev-interaction trapped protein 1 (KRIT1) is an endothelial scaffolding protein that associates with the adherens junction (AJ) (DiStefano et al., 2014; Glading and Ginsberg, 2010; Glading et al., 2007; Lisowska et al., 2018). Loss of KRIT1 expression leads to the development of cerebral cavernous malformations (CCM) (Del Curling et al., 1991; Zabramski et al., 1994), abnormalities characterized by leaky, enlarged (‘cavernous’) microvascular lesions in the brain and other tissues (Chan et al., 2010; Clatterbuck, 2001; Revencu, 2006). Recent evidence suggests that KRIT1 plays an important role in limiting the endothelial response to inflammation and maintaining endothelial quiescence (Antognelli et al., 2018; Corr et al., 2012; Glading and Ginsberg, 2010; Goitre et al., 2014, 2017). Although much is known about the impact of loss of KRIT1 expression on endothelial cell signaling and behavior, advancement of the field is hindered by a lack of knowledge about how KRIT1 is itself regulated, which is not only critical to understanding the clinical impact of future treatments for CCM, but important to understanding the mechanisms that limit endothelial activation.

**Fig. 1.**
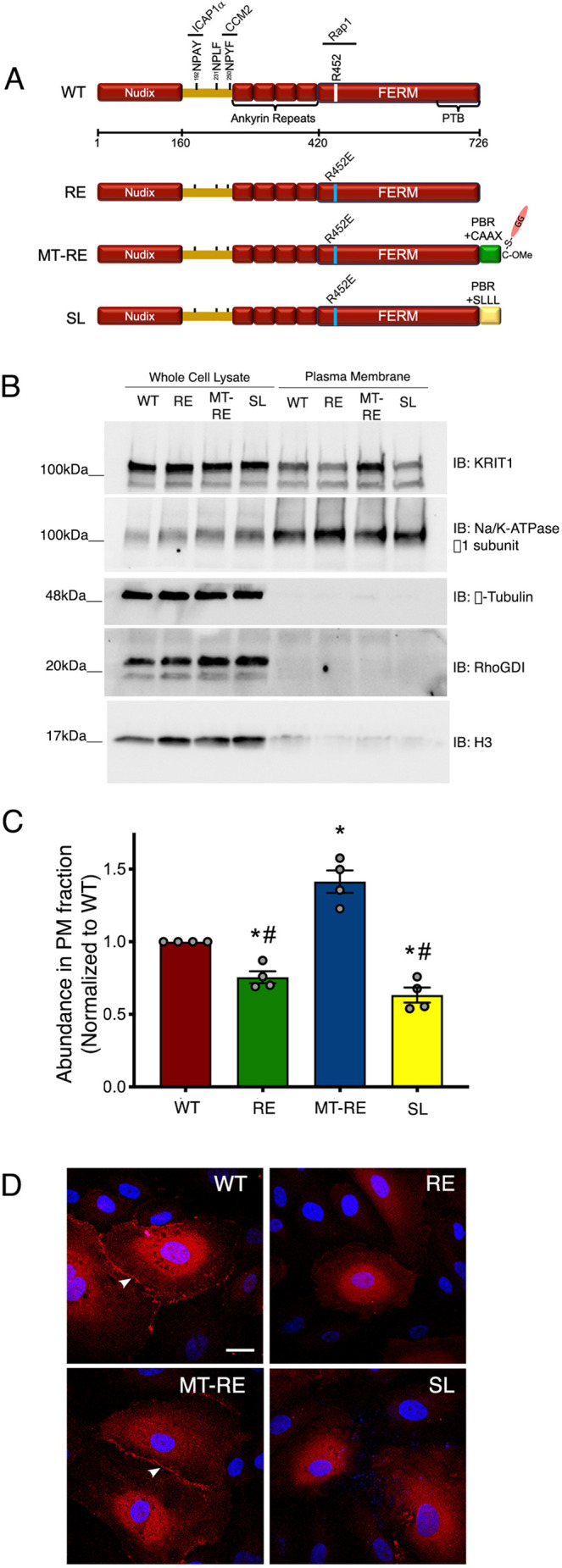
**Lipid modification of KRIT1 promotes membrane localization independently of Rap1 binding.** (A) Diagram of mCherry-tagged KRIT1 constructs used in this study. Sites for ICAP1α and CCM2 interactions are noted at the NPxY/F motifs. R452E mutation was introduced in the FERM domain. C-terminal geranylgeranylation (GG) was achieved by addition of Rap1a polybasic region (PBR) and CAAX box (CLLL) to the C-terminus of KRIT1-R452E. As a lipidation control, the CAAX sequence was mutated to SAAX (SL). (B) HEK293 cells transfected with mCherry-KRIT1 constructs were separated into subcellular fractions. Plasma membrane (PM) fractions were isolated and western blotted for KRIT1, as well as Na^+^/K^+^-ATPase (PM marker), RhoGDI (cytosolic marker), and histone H3 (nuclear marker). Protein content was measured in both total and PM fractions to ensure equal loading across samples. Top KRIT1 bands correspond to mCherry-tagged constructs, and bottom bands are endogenous KRIT1. Blots are representative of *n*=4 independent experiments. IB, immunoblot. (C) Densitometry quantification of PM fractions in B. Data shown are mean normalized band densities±s.e.m., normalized to WT, *n*=4. *P<0.05 by Tukey post-hoc testing vs WT. ^#^*P*<0.001 by Tukey post-hoc testing vs MT-RE. *P*<0.0001 by one-way ANOVA. (D) HPAEC depleted of endogenous KRIT1 were adenovirally transduced with mCherry-KRIT1 constructs (red). Nuclei were labeled with Hoechst 33258 and are shown in blue. WT and MT-RE constructs localized to points of cell–cell contact (arrowheads), whereas RE and SLLL were primarily cytoplasmic. Representative confocal images from 10–12 fields per construct across *n*=5 independent experiments. Scale bar: 25 µm.

KRIT1 contains several protein-interacting domains, allowing it to associate with a number of binding partners (Fig. 1A). Previous work has established that the interaction of the KRIT1 FERM domain with the small GTPase Rap1 is important for KRIT1-mediated junction stabilization (Glading et al., 2007). This interaction promotes KRIT1 localization to cell–cell contacts and association with the AJ protein β-catenin, concomitant with stabilization of the endothelial barrier (Glading et al., 2007; Liu et al., 2011). In addition, it has been shown that NPxY/F motifs in the relatively unstructured N-terminal half of KRIT1 are the sites of interaction with ICAP1α, a negative regulator of β1 integrin (Béraud-Dufour et al., 2007; Faurobert et al., 2013; Liu et al., 2013; Zawistowski et al., 2005) and CCM2, which associate with KRIT1-^192^NPAY^195^ and KRIT1-^250^NPYF^253^, respectively (Béraud-Dufour et al., 2007; Fisher et al., 2015; Liu et al., 2013) (Fig. 1A). The KRIT1-^192^NPAY^195^ motif has also been reported to be the site of a putative intramolecular interaction between the KRIT1 N-terminus and KRIT1's C-terminal phosphotyrosine-binding (PTB) domain (Béraud-Dufour et al., 2007; Francalanci et al., 2009). This putative intramolecular interaction has been hypothesized to create an autoregulatory ‘closed’ conformation similar to that observed in other FERM-domain-containing proteins (Goksoy et al., 2008; Goult et al., 2009; Li et al., 2007; Pearson et al., 2000). Binding of ICAP1α has been suggested to relieve this intramolecular interaction because of competition for the KRIT1-^192^NPAY^195^ motif, but other binding proteins, such as CCM2 or Rap1, could also inhibit this interaction via steric hindrance (Béraud-Dufour et al., 2007; Francalanci et al., 2009).

Although these studies have laid out a framework for understanding the role of KRIT1 as a junctional scaffold, we do not clearly understand how these interactions influence the physiological function of KRIT1 as a regulator of AJ stability and endothelial barrier function (DiStefano et al., 2014; Glading et al., 2007; Lisowska et al., 2018; Liu et al., 2011). As stated above, binding of active Rap1 to KRIT1 promotes its junctional localization. KRIT1, in turn, is required for the ability of Rap1 activation to stabilize cell–cell contacts, as activation of Rap1 after KRIT1 depletion is not sufficient to rescue thrombin-mediated disruption of endothelial barrier function (Glading et al., 2007). Disruption of KRIT1 binding to Rap1 via a point mutation in the KRIT1 FERM domain (R452E) results in loss of KRIT1 localization with AJ and disrupted barrier function (Li et al., 2012; Liu et al., 2011). However, loss of microtubular structure allowed KRIT1-R452E to colocalize with VE-cadherin at endothelial cell–cell contacts (Liu et al., 2011), though the loss of cytoskeletal integrity precluded subsequent analysis of the effect on the endothelial barrier. Furthermore, although it is well established that the KRIT1 N-terminus competes with β1 integrin for binding to ICAP1α (Liu et al., 2013), and although the KRIT1–ICAP1α interaction has been suggested to be important for nuclear accumulation of both proteins (Draheim et al., 2017), it is unknown how the interaction with ICAP1α affects the ability of KRIT1 to stabilize cell–cell contacts.

The lack of knowledge about how these binding interactions control the physiological function of KRIT1, specifically in the context of full-length KRIT1 protein, prompted us to undertake a systematic analysis of the contribution of Rap1 and ICAP1α binding to KRIT1 junctional localization and barrier stability. Therefore, we created a series of fluorescently labeled KRIT1 mutants defective in either Rap1 binding (KRIT1-R452E, herein referred to as RE), ICAP1α binding (KRIT1 ^192^NPAY^195^ to ^192^APAA^195^, herein referred to as APAA), or bearing a disrupted PTB domain, alone or in combination. In addition, we tested whether these interactions acted by altering the junctional localization of KRIT1 by directly targeting our mutants to the plasma membrane via lipid modification. We then used this panel of constructs to assess which interactions were critical to the ability of KRIT1 to promote junctional localization of the AJ protein β-catenin, stabilize endothelial barrier function and regulate β1 integrin activation. Surprisingly, our results suggest that loss of the intramolecular interaction between the N- and C-termini of KRIT1 is the critical mechanism leading to stabilization of the endothelial barrier, irrespective of whether KRIT1 is cytoplasmic or junctionally localized, and implicate for the first time a significant role for β1 integrin in regulating this phenomenon.

## RESULTS

### Membrane-targeted KRIT1 is enriched at endothelial cell–cell contacts

In confluent endothelial cells, a proportion of KRIT1 is localized to the plasma membrane at sites of cell–cell contact (Gingras et al., 2012; Glading et al., 2007; Glading and Ginsberg, 2010; Liu et al., 2011). We have previously shown that activation of Rap1 increases the localization of KRIT1 to cell–cell contacts, which correlates with an increase in the stability of VE-cadherin and the junctional complex (Glading et al., 2007; Liu et al., 2011). Although KRIT1 contains an ankyrin-repeat domain that has been hypothesized to stabilize the interaction of KRIT1 with phosphoinositides in the membrane, KRIT1 is not considered to be directly targeted to the plasma membrane in the absence of Rap1. Indeed, Béraud-Dufour et al. showed that although KRIT1 associates with PIP2 independently of Rap1, association with PIP2 is enhanced by Rap1 binding to KRIT1 (Béraud-Dufour et al., 2007). A previous study suggested that the membrane localization of KRIT1 is limited by its association with the microtubule cytoskeleton, and that disruption of the microtubule cytoskeleton enables KRIT1 to localize to cell–cell contacts in the absence of Rap1 binding (Liu et al., 2011). However, owing to the massive disruption of cytoskeletal integrity, it is unclear whether membrane localization of KRIT1 is sufficient to maintain endothelial barrier function in these conditions.

As we wanted to distinguish between a specific role for Rap1 binding vs membrane localization in the regulation of KRIT1 function, we directly targeted fluorescently tagged (mCherry) KRIT1 to the plasma membrane by attaching the C-terminal polybasic region and CAAX box (KKKPKKKSCLLL) of Rap1 to the C-terminus of KRIT1. In concert, we introduced the point mutation R452E, which decreases Rap1 binding to KRIT1, resulting in a membrane-targeted and Rap1-binding-deficient KRIT1 (referred to herein as MT-RE). As a negative control, the cysteine of the CAAX box was mutated to serine to prevent lipid modification at this site (SL, Fig. 1A). Addition of the intact or mutant lipid modification site had no effect on the decreased Rap1 binding resulting from the R452E mutation (Fig. S1A,B). Next, we examined whether MT-RE exhibited significant membrane localization using subcellular fractionation. Plasma membrane fractions were isolated from HEK293A cells transiently transfected with our KRIT1 constructs and probed for KRIT1. KRIT1-RE exhibited significantly reduced abundance in the plasma membrane fraction compared to wild type (WT) (Fig. 1B,C), consistent with previous reports of the primarily cytosolic localization of KRIT1-R452E (Liu et al., 2011). However, membrane-targeting of RE enriched the abundance of mCherry-KRIT1 in the plasma membrane fraction, supporting the conclusion that MT-RE is membrane targeted.

Having confirmed biochemically that the addition of the Rap1 targeting sequence had the desired effect, we next assessed the subcellular localization of our KRIT1 constructs in endothelial cells using confocal microscopy. Human pulmonary artery endothelial cells (HPAEC) were first transduced with lentivirus encoding anti-KRIT1 shRNA, which reduced endogenous KRIT1 expression by ∼65% compared to scramble shRNA control (scr, Fig. S2A,B). Twenty-four hours later, KRIT1-shRNA-expressing cells were transduced with adenovirus containing fluorescently tagged WT, RE, MT-RE and SL KRIT1 at levels roughly 12–15-fold higher than endogenous KRIT1 (Fig. S2C). As endogenous KRIT1 is expressed at low levels in endothelial cells, this overexpression was necessary in order to yield signal sufficient for imaging. Eighteen hours after adenovirus transduction, cells were fixed and processed for imaging. Subsequent laser scanning confocal microscopy indicated that RE and SL constructs remained primarily cytosolic, in agreement with previously published work (Liu et al., 2011), whereas both WT and MT-RE localized to and were enriched at cell–cell contacts (Fig. 1D, arrowheads).

### Membrane-targeted KRIT1-R452E partially rescues β-catenin localization at cell–cell contacts

**Fig. 2.**
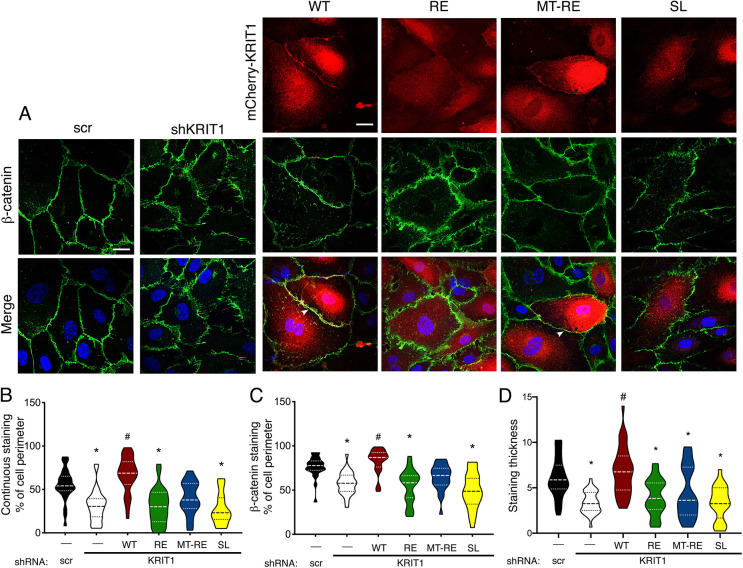
**Membrane-targeted KRIT1 localizes to points of cell–cell contacts independently of Rap1 binding while partially rescuing AJ stability.** (A) HPAEC depleted of endogenous KRIT1 (shKRIT1) were adenovirally transduced with m-Cherry tagged KRIT1 constructs (red) and stained for β-catenin (green). Nuclei were labeled with Hoechst 33258 and are shown in blue in merge images. WT and MT-RE constructs localized to cell–cell contacts and promoted β-catenin staining at the cell border (arrowheads). Representative confocal images from 10–12 fields per construct across *n*=5 independent experiments. Scale bars: 25 µm. (B) Quantification of β-catenin staining continuity and (C) coverage at the cell perimeter was calculated by the Junction Analysis Program (JAnaP), and junction width (D) was calculated using ImageJ. Violin plots represent data point frequency distribution between minimum and maximum values, with quartiles indicated by dotted lines, and median indicated by a dashed line. Data shown are from *n*=23–35 cells from five independent experiments. *P<0.001 by Bonferroni post-hoc testing vs scramble shRNA alone (scr). #*P*<0.001 by Bonferroni post-hoc testing vs shKRIT1 alone. *P*<0.0001 by one-way ANOVA.

Having established that MT-RE was able to traffic to sites of cell–cell contact and had reduced binding to Rap1, we next wanted to examine whether MT-RE could rescue AJ protein localization in KRIT1-depleted cells. We were specifically interested in the localization of β-catenin, which we previously showed dissociates from VE-cadherin in the absence of KRIT1, and subsequently promotes β-catenin-mediated transcription (Glading and Ginsberg, 2010). Immunostaining of β-catenin in HPAEC transduced with lentiviral KRIT1 shRNA (shKRIT1) demonstrated the expected disrupted pattern compared to scrambled shRNA control (scr, Fig. 2A) (Glading et al., 2007; Glading and Ginsberg, 2010; Lisowska et al., 2018; Liu et al., 2011). Re-expression of WT KRIT1 rescued β-catenin localization and distribution, as well as significant co-localization of β-catenin and KRIT1 staining (arrowheads, Fig. 2A). However, expression of RE or SL was unable to rescue the disrupted β-catenin staining in KRIT1-depleted cells. MT-RE appeared partially able to restore the level and pattern of β-catenin staining, though it showed similar colocalization with β-catenin when compared to WT KRIT1 (Fig. 2A). To directly quantify the contiguity and coverage of the β-catenin staining across many fields, we utilized the junction analyzer program JAnaP (Gray et al., 2019). Continuous β-catenin staining at the cell border, defined as any positively stained segment over ≥4.3 µm (15 pixels) in length, was reduced by 24% after depletion of endogenous KRIT1. Re-expression of WT KRIT1 restored β-catenin continuity, but RE and SL failed to do so (30.4% of cell perimeter for RE vs 66.9% for WT, Fig. 2B). Interestingly, although cells rescued with MT-RE had reduced continuous β-catenin staining (Fig. 2B), this condition was not significantly different from either scramble or shKRIT1 controls, supporting a partial rescue of AJ stability. Assessment of total cell perimeter coverage of β-catenin staining revealed a similar trend, albeit with smaller differences between disrupted and rescued conditions (Fig. 2C). However, measurement of β-catenin staining thickness, perpendicular to the cell edge, indicated that MT-RE expression yielded similar junctional thickness as RE (Fig. 2D). This suggests that perturbed β-catenin staining is not simply the result of loss of total β-catenin at the AJs, but is caused by changes in the junctional integrity.

### Membrane-targeted KRIT1 is not sufficient to rescue barrier function after inhibition of Rap1 binding

**Fig. 3.**
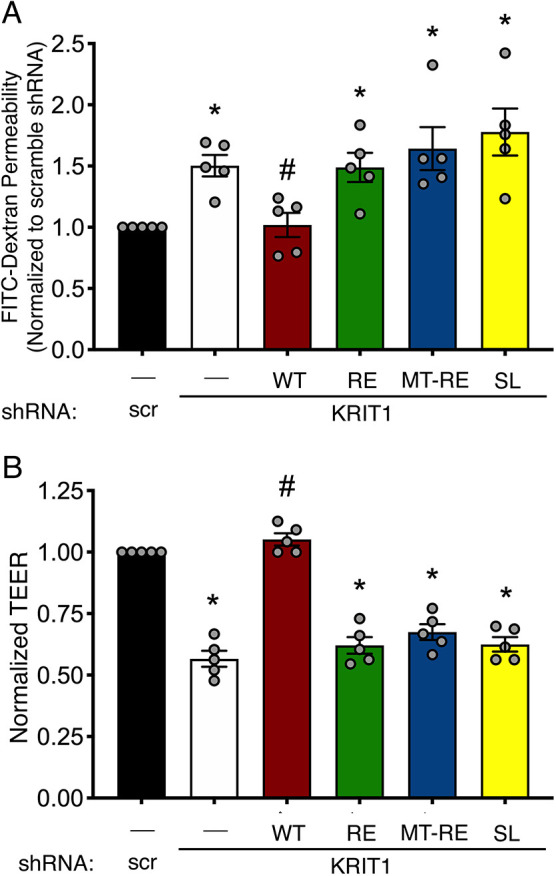
**Membrane-targeted KRIT1-RE does not rescue endothelial barrier function.** (A) Permeability of transduced monolayers to 40 kDa FITC-dextran. Data shown are mean permeability±s.e.m., normalized to scramble shRNA alone (scr), from *n*=5 independent experiments. (B) TEER of confluent HPAEC monolayers. Resistance reading of an empty FN-coated Transwell was subtracted from resistance values of Transwells containing HPAEC, then multiplied by the growth area of the wells, yielding Ω*cm^2^ values. Data shown are mean Ω*cm^2^ values±s.e.m., normalized to scramble shRNA alone (negative control). *P<0.05 by Tukey post-hoc testing vs scramble shRNA alone. #*P*<0.05 by Tukey post-hoc testing vs shKRIT1 alone. *P*<0.0001 by one-way ANOVA.

Changes in AJ stability in endothelial cells depleted of KRIT1 correlate with a loss of barrier function (Glading et al., 2007; Liu et al., 2011), which is thought to drive much of the clinical phenotype of CCM. Given that MT-RE localized to HPAE cell–cell contacts and appeared to partially rescue AJ stability, we next wanted to test whether membrane-targeted KRIT1 could rescue endothelial barrier function in the absence of Rap1 binding. We again transduced our cells with lentivirus encoding either scramble shRNA or shKRIT1. However, to limit potential overexpression artifacts in our functional assays, we lowered the adenoviral multiplicity of infection (MOI) so that our mCherry-KRIT1 constructs were expressed at roughly 1.5-fold endogenous KRIT1 (Fig. S3). HPAEC were seeded on 3-µm pore size polyester Transwell™ inserts after lentiviral shRNA transduction but before being transduced with adenoviral KRIT1 constructs, then allowed to form a confluent monolayer. Barrier function was assessed by measuring the permeability of the monolayer to 40-kDa FITC-dextran (Fig. 3A) and by measuring transendothelial electrical resistance (TEER; Fig. 3B). Both methods demonstrated that KRIT1 depletion resulted in loss of the barrier as indicated by an ∼50% increase in dextran permeability and an ∼50% decrease in electrical resistance of the monolayer, compared to scramble shRNA control. Expression of WT KRIT1 rescued barrier function to levels similar to scramble shRNA control; expression of RE and SL were unable to rescue barrier function as measured by either method. Surprisingly, expression of MT-RE failed to rescue endothelial barrier function, despite partially rescuing β-catenin localization. This suggests that the presence of KRIT1 at sites of cell–cell contact is insufficient to stabilize the endothelial barrier.

### Mutation of ^192^NPAY^195^ in KRIT1 reduces binding to ICAP1α but not CCM2

Previous studies have indicated that the ^192^NPAY^195^ motif is an important regulatory site on KRIT1. Mutation of this site in KRIT1 peptide fragments blocks binding to ICAP1α, reducing β1 integrin activation (Liu et al., 2013), and inhibits binding between C-terminal and N-terminal fragments of KRIT1 (Béraud-Dufour et al., 2007; Francalanci et al., 2009). To test the contribution of this regulatory site to the barrier-promoting function of KRIT1, we mutated ^192^NPAY^195^ to APAA in both our WT and RE-containing KRIT1 constructs. In addition, we postulated that the inability of MT-RE KRIT1 to rescue barrier function could be caused by the effect of Rap1 binding on KRIT1 self-association, rather than a requirement for Rap1 per se; therefore, we also introduced the APAA mutation into this construct. We confirmed that all APAA-RE-containing constructs exhibited decreased interaction with Rap1 by pulldown (Fig. S4).

**Fig. 4.**
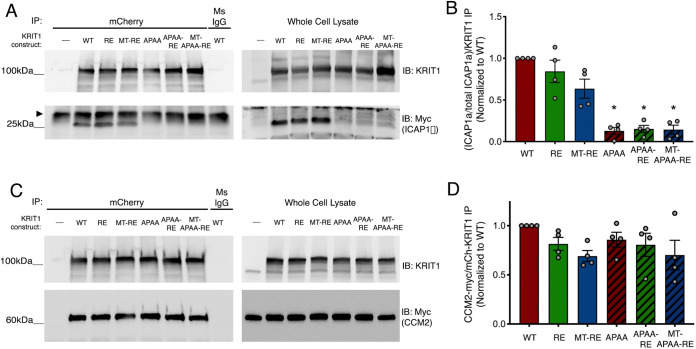
**KRIT1-APAA mutation inhibits association with ICAP1α but not CCM2.** (A) mCherry-KRIT1 constructs were immunoprecipitated (IP) from HEK293A lysates co-transfected with ICAP1α -Myc and blotted (IB) for KRIT1 (upper blots) and ICAP1α-Myc (lower blots). Arrowhead indicates nonspecific band present in all lanes of IP. In whole cell lysate (input) KRIT1 blot, upper bands correspond to mCherry-tagged constructs, and bottom bands are endogenous KRIT1. Blots were stripped and reprobed for Myc. Blots are representative of *n*=5 independent experiments. Ms IgG, non-immune mouse IgG. (B) Quantification of ICAP1α-Myc band density in A, relative to respective ICAP1α-Myc expression and KRIT1 IP, normalized to WT KRIT1. Data shown are mean normalized band densities±s.e.m. *P<0.0001 by Tukey post-hoc testing vs WT. *P*=0.0003 by one-way ANOVA. (C) mCherry-KRIT1 constructs were immunoprecipitated from HEK293A lysates co-transfected with CCM2-Myc and blotted for KRIT1 (upper blots) and CCM2-Myc (lower blots). Blots are representative of *n*=4 independent experiments. (D) Quantification of CCM2-Myc band density in C. Data shown are mean normalized band densities±s.e.m., *n*=4 independent experiments. No statistical significance by one-way ANOVA.

To verify the effects of the APAA mutation on ICAP1α binding to full-length KRIT1, we transfected HEK293 cells with ICAP1α-Myc in combination with each of our KRIT1 constructs. Lysates were immunoprecipitated with anti-mCherry to pull down the exogenous KRIT1 and blotted for Myc to detect ICAP1α. Interestingly, WT, RE and MT-RE conditions contained 4-fold more ICAP1α compared to expression of ICAP1α alone, whereas expression of APAA constructs exhibited ICAP1α expression at levels similar to control (Fig. 4A), consistent with previous reports (Faurobert et al., 2013). To account for this, densitometric analysis of coimmunoprecipitated ICAP1α was normalized to the respective whole cell lysate control, and this was then normalized to the amount of immunoprecipitated mCherry-KRIT1 for each condition (Fig. 4B). APAA-KRIT1, APAA-R425E KRIT1 and MT-APAA-RE KRIT1 all showed reduced binding to ICAP1α compared to controls, even when the reduction in ICAP1α expression was taken into account (Fig. 4A,B). In parallel, we also assessed binding of our constructs to CCM2, another protein involved in the formation of CCM, which has also been suggested to regulate KRIT1 subcellular localization and function (Fisher et al., 2015; Lisowska et al., 2018; Stockton et al., 2010; Zawistowski et al., 2005; Zhang et al., 2007). mCherry-KRIT1 constructs were immunoprecipitated from HEK293A cells co-transfected with CCM2-Myc. Samples were western blotted, and CCM2-Myc bands were normalized to their respective immunoprecipitated mCherry-KRIT1 band to evaluate the relative extent of interaction. Densitometry analysis revealed no statistically significant change in association of any KRIT1 constructs with CCM2, compared to WT (Fig. 4C,D), indicating that changes in the KRIT1-CCM2 interaction were not a likely determinant of KRIT1 function in our model. This conclusion was also supported by our examination of *Klf2* mRNA levels in HPAEC (Fig. S5). Consistent with previous reports, loss of endogenous KRIT1 increased *Klf2* mRNA transcription (Lopez-Ramirez et al., 2017; Renz et al., 2015; Zhou et al., 2015, 2016). Only re-expression of WT or MT-RE at physiological levels resulted in a statistically significant rescue, albeit not to the baseline levels seen in scramble control. Interestingly, APAA-mutated constructs displayed similar *Klf2* mRNA expression as KRIT1-RE. Thus, there was no discernible trend in *Klf2* expression that correlated with changes in junctional protein localization and barrier stability, suggesting that *Klf2* expression does not play a significant role in this context.

### Mutation of ^192^NPAY^195^ in KRIT1 rescues AJ stability and endothelial barrier function

**Fig. 5.**
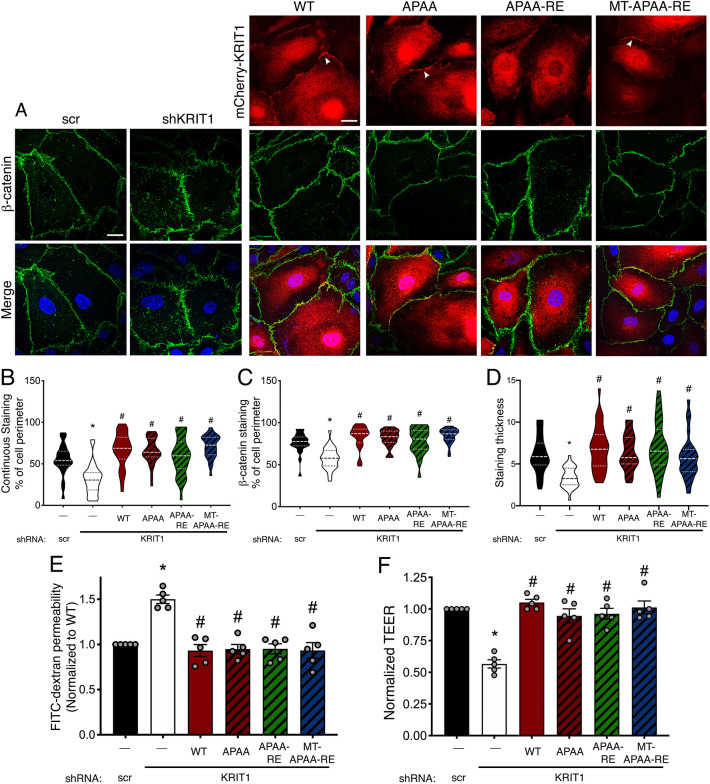
**KRIT1-APAA mutation promotes stable barrier function independently of Rap1 binding or junctional localization.** (A) HPAEC depleted of endogenous KRIT1 (shKRIT1) were adenovirally transduced with KRIT1 constructs (red) and then stained for β-catenin (green). Nuclei were labeled with Hoechst 33258 and are shown in blue in merge images. WT, APAA, and MT-APAA-RE constructs localized to points of cell–cell contact (arrowheads), whereas APAA-RE was primarily cytoplasmic. Representative confocal images from 10–12 fields per construct across *n*=5 independent experiments. Scale bars: 25 µm. (B–D) Quantification of β-catenin staining continuity (B), coverage at the cell perimeter (C), and junction width (D). Violin plots represent data point frequency distribution between minimum and maximum values, with quartiles indicated by dotted lines, and median indicated by a dashed line. Data shown are from *n*=26–34 cells from five independent experiments. *P<0.001 by Bonferroni post-hoc testing vs scramble shRNA alone. #*P*<0.001 by Bonferroni post-hoc testing vs shKRIT1 alone. *P*<0.0001 by one-way ANOVA. (E) Permeability of 40 kDa FITC-dextran in transduced confluent HPAEC monolayers. Data shown are mean permeability±s.e.m., normalized to scramble shRNA alone (negative control), from *n*=5 independent experiments. *P<0.05 by Tukey post-hoc testing vs scramble shRNA alone. #*P*<0.05 by Tukey post-hoc testing vs shKRIT1 alone. *P*<0.0001 by one-way ANOVA. (F) TEER of confluent HPAEC monolayers per unit area. Data shown are mean Ω*cm^2^ values±s.e.m., normalized to scramble shRNA alone (scr). *P<0.05 by Tukey post-hoc testing vs scramble shRNA alone, from *n*=5 independent experiments. #*P*<0.05 by Tukey post-hoc testing vs shKRIT1 alone. *P*<0.0001 by one-way ANOVA.

Next, APAA-containing constructs were overexpressed in HPAEC depleted of endogenous KRIT1 and imaged using confocal microscopy. WT KRIT1, APAA and MT-APAA-RE were similarly enriched at cell–cell contacts (Fig. 5A, arrowheads). Mutation of the NPxY motif did not significantly alter the localization of KRIT1-RE (Fig. 5A, APAA-RE), which remained cytosolic. However, assessment of β-catenin staining in these cells indicated that all constructs with the APAA mutation were able to rescue β-catenin localization and distribution, suggesting that they promote junction stability. This was confirmed by quantification using JAnaP to assess β-catenin staining continuity (Fig. 5B), coverage (Fig. 5C) and width (Fig. 5D). This quantification indicated that β-catenin staining was restored to levels similar to WT KRIT1 in cells expressing any construct containing the APAA mutation, including APAA-RE, which did not localize to junctions. This was particularly interesting considering that KRIT1 localization at cell–cell contacts has been assumed to be necessary for its junction-stabilizing effect (Glading et al., 2007; Glading and Ginsberg, 2010; Liu et al., 2011).

Next, we examined how the presence of the APAA mutation affected the ability of KRIT1 to rescue endothelial barrier function in KRIT1-depleted cells. Both flux of 40 kDa FITC-dextran and TEER were restored to levels similar to scrambled shRNA control when KRIT1-depleted cells were rescued with WT KRIT1, APAA, APAA-R425E and MT-APAA-RE (Fig. 5E,F). As both WT KRIT1, which binds ICAP1α, and the APAA mutants, which do not bind ICAP1α, rescued barrier function, these results strongly suggest that mutation of ^192^NPAY^195^ is sufficient to rescue barrier function in the absence of Rap1 binding, ICAP1α binding or junctional enrichment.

### Mutation of W688 in KRIT1 rescues AJ stability and endothelial barrier function

The ^192^NPAY^195^ motif mediates ICAP1α binding, but is also required for the binding of KRIT1's C-terminal PTB domain to the N-terminal half of KRIT1 (Béraud-Dufour et al., 2007). As we inferred from the data above that ICAP1α was not required for the ability of KRIT1 to stabilize the endothelial barrier, we wanted to more directly test the requirement for an N-to-C terminal interaction. Previous studies used structural modeling to identify the NPXY binding cleft in the KRIT1 PTB domain, which is formed between a β-sheet (residues 686–690) and an α-helix (residues 714–728) (Francalanci et al., 2009). As disruptions to these structured domains could affect protein folding and stability, we first verified protein folding using circular dichroism (CD) of WT and mutated FERM domain fragments. Introduction of multiple mutations in the β-sheet or truncation of the PTB domain resulted in proteins that were resistant to purification, were poorly expressed in mammalian cells and were sensitive to degradation by the proteasome, and failed to bind ICAP1α despite the presence of an intact NPxY motif (data not shown), suggesting that substantial alterations to the PTB domain leads to improper folding of KRIT1. Therefore, we made a single conservative mutation in the middle of the β-sheet (W688A), which was predicted to disrupt the interaction with NPXY by molecular modeling. CD of the W688A mutant FERM domain indicated only minimal disruption of folding in this region, with a slight reduction in regular helical content, after W688A mutation (Fig. S6A,B).

**Fig. 6.**
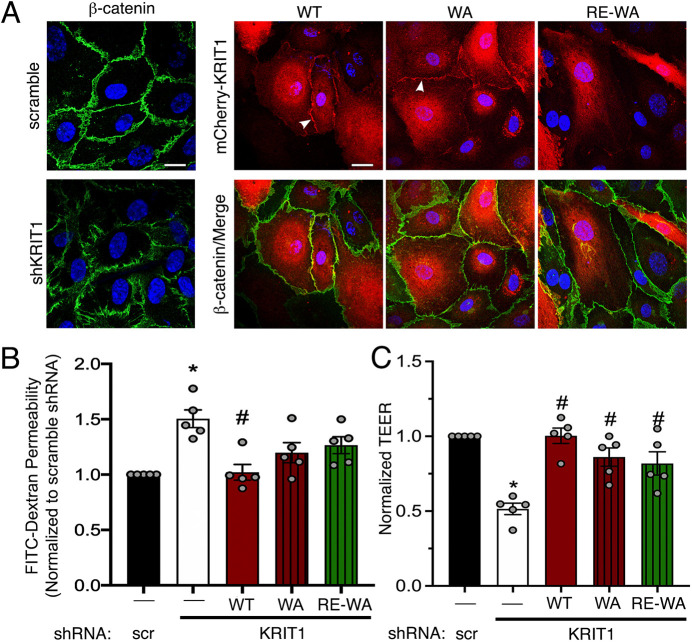
**W688A mutation rescues barrier function independently of Rap1 binding or junction localization.** (A) HPAEC depleted of endogenous KRIT1 were adenovirally transduced with mCherry-KRIT1 constructs (red). WT and WA constructs localized to points of cell–cell contact (arrowheads), whereas RE-WA was primarily cytosolic. β-catenin immunostaining was carried out to mark the cell border (green). Nuclei were labeled with Hoechst 33258 and are shown in blue. Representative confocal images from 10 fields per construct across *n*=4 independent experiments. Scale bars: 25 µm. (B) Permeability of 40 kDa FITC-dextran in transduced confluent HPAEC monolayers. Data shown are mean permeability±s.e.m., normalized to scramble shRNA alone (scr, negative control), from *n*=5 independent experiments. *P<0.05 by Tukey post-hoc testing vs scramble shRNA alone. #*P*<0.05 by Tukey post-hoc testing vs shKRIT1 alone. *P*<0.0001 by one-way ANOVA. (C) TEER of confluent HPAEC monolayers per unit area. Data shown are mean Ω*cm^2^ values±s.e.m., normalized to scramble shRNA alone. *P<0.05 by Tukey post-hoc testing vs scramble shRNA alone, from *n*=5 independent experiments. #*P*<0.05 by Tukey post-hoc testing vs shKRIT1 alone. *P*<0.0001 by one-way ANOVA.

We then mutated W688A in full-length KRIT1, both in the presence and absence of the R452E mutation (WA and RE-WA, respectively). These constructs were adenovirally overexpressed in HPAEC depleted of endogenous KRIT1 at roughly 10× endogenous levels (Fig. S6C,D). Imaging revealed that WA localized to the cell edge, similar to WT. Addition of the R452E mutation once again inhibited localization to cell–cell contacts (Fig. 6A). Next, these constructs were expressed at ∼1.5× endogenous levels (Fig. S6E,F) in HPAEC depleted of endogenous KRIT1 in order to evaluate barrier function. Both WA and RE-WA constructs partially rescued FITC-dextran permeability, as the average FITC-dextran leak in these conditions was not significantly different from either scramble or KRIT1 shRNA conditions (Fig. 6A). On the other hand, the WA and RE-WA mutant KRIT1 was able to rescue permeability as measured by TEER (Fig. 6C), suggesting that conservative modification of the KRIT1 PTB can restore barrier function. As shown for the APAA mutation, both constructs yielded similar barrier stability regardless of localization, again supporting the idea that KRIT1 might be able to stabilize endothelial barriers independently of Rap1 binding or junctional localization.

### Rescue of barrier function by KRIT1 mutant proteins correlates with changes in β1-integrin-mediated adhesion

The discovery that disruption of the ^192^NPAY^195^ motif abrogates the need for ICAP1α or Rap1 binding, or even junctional localization, to allow KRIT1 to promote endothelial barrier function, suggests that our current understanding of how KRIT1 stabilizes AJ is incomplete. We noted that the ^192^NPAY^195^ motif has been shown to regulate KRIT1's intramolecular interaction, but that it also appears to be important for the ability of KRIT1 to modify β1 integrin activity. Liu et al. reported that the cells expressing KRIT1-APAA exhibit lower levels of integrin activation (Liu et al., 2013), which they attributed to the loss of the interaction between KRIT1 and ICAP1α and subsequent increased binding of ICAP1α to β1 integrins. Conversely, Faurobert et al. reported that loss of KRIT1 or CCM2 expression led to an increase in β1 integrin activity concomitant with degradation of ICAP1α protein (Faurobert et al., 2013). Despite the focus on the role of ICAP1α in these studies, we considered that β1 integrin activation could potentially contribute to KRIT1-mediated regulation of barrier function, as knockdown of β1 integrin in endothelial cells depleted of KRIT1 has been shown to trigger a decrease in stress fibers and an increase in cortical actin, a shift known to be associated with barrier stabilization (Faurobert et al., 2013).

**Fig. 7.**
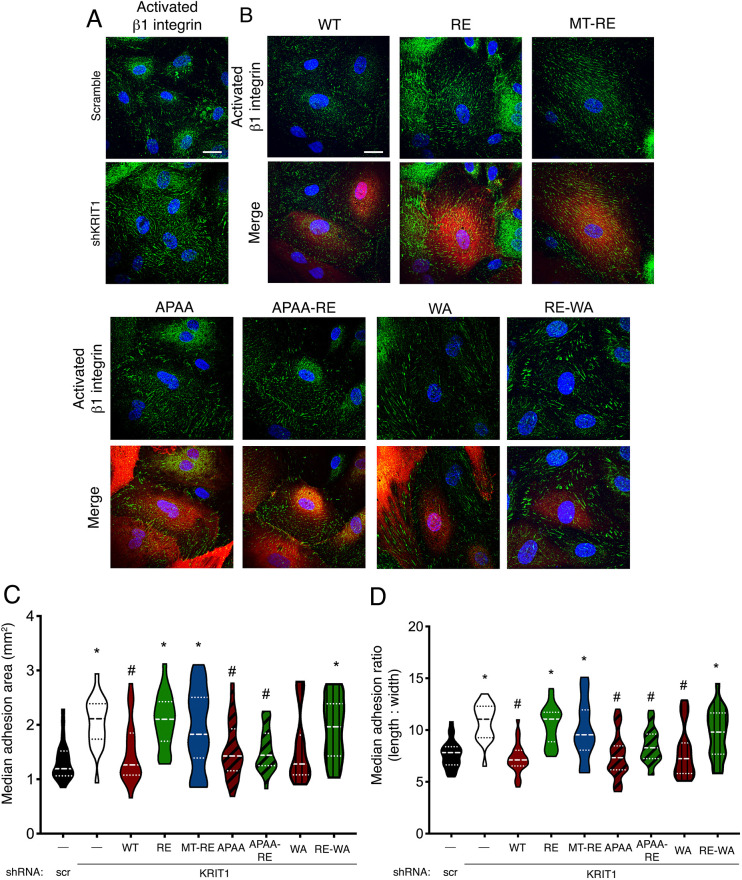
**KRIT1 constructs that rescue barrier function limit β1-integrin-containing adhesion size and promote peripheral focal contacts.** (A) HPAEC were transduced with scramble or KRIT1-targeting shRNA (shKRIT1) alone and immunostained with a β1 integrin activation-sensitive antibody (green). Nuclei were labeled with Hoechst 33258 and are shown in blue. Representative confocal images from 22 or 23 fields per condition across *n*=5 independent experiments. Scale bar: 25 µm. (B) HPAEC depleted of endogenous KRIT1 were adenovirally transduced with mCherry-KRIT1 constructs (red signal in merge images) and immunostained for active β1 integrin (green). Nuclei were labeled with Hoechst 33258 and are shown in blue. Representative confocal images from 20–25 fields per construct across *n*=5 independent experiments. Scale bar: 25 µm. (C,D) β1-integrin-containing adhesion area (C) and length:width ratio (D) was calculated using Imaris image analysis software. Violin plots represent data point frequency distribution between minimum and maximum values, with quartiles indicated by dotted lines, and median indicated by a dashed line. Data shown are from *n*=20–25 cells from five independent experiments. Overall *P*<0.0001 by one-way ANOVA; *P<0.05 vs scramble shRNA, #*P*<0.05 vs shKRIT1 by Bonferroni post-hoc testing.

We examined how expression of our constructs affected β1 integrin activation in HPAEC by immunostaining with an antibody specific for active β1 integrin (HUTS-4, Luque et al., 1996). Whereas total β1 integrin expression was similar in all conditions (Fig. S7A–D), knockdown of KRIT1 increased the appearance of active β1-integrin-containing fibrillar adhesion structures throughout the cell body. In contrast, scramble shRNA control samples typically exhibited small, focal, active β1-containing structures located near the cell edge (Fig. 7A). Rescue of KRIT1 expression with WT KRIT1 reversed the fibrillar adhesion phenotype and restored the appearance of peripheral adhesions. In contrast, expression of RE or MT-RE in KRIT1-depleted cells failed to reverse the development of fibrillar adhesions. However, expression of any of the APAA-containing constructs or the WA mutation blocked the formation of central fibrillar adhesions, and promoted peripheral focal contacts, similar to cells rescued with WT KRIT1 (Fig. 7B). As MT-RE-APAA yielded similar results to other APAA-containing constructs, this condition was not shown. Interestingly, rescue with RE-WA KRIT1 resulted in slightly larger peripheral adhesions (Fig. 7B).

To quantify these observations across multiple fields and replicates, HUTS-4-positive adhesions within individual cells were identified and analyzed using Imaris image analysis software. The total number of adhesions, total area of adhesion and average adhesion length:width ratio were calculated for each cell. Although there was no significant difference in the number of HUTS-4-positive adhesions in each condition (Fig. S7E), total adhesion area was higher in shKRIT1-, RE- and MT-RE-expressing cells (Fig. 7C), which correlated with the inability of these constructs to rescue barrier function. These conditions also exhibited a higher median fibril length:width ratio (e.g. 10.80±0.39 for shKRIT1 vs 7.25±0.30 for WT rescue, Fig. 7D), confirming the presence of more fibrillar adhesion structures. Conversely, KRIT1-shRNA-transduced cells rescued with WT, WA or all APAA-containing constructs (APAA, APAA-RE) displayed reduced total adhesion area and a lower median fibril length:width ratio, comparable to scramble-shRNA-transduced cells. As this was also observed in conditions where KRIT1 does not localize to cell–cell contacts, it is possible that junctional and barrier stabilization occur via KRIT1-mediated indirect regulation of β1 integrin.

### Mutation of ^192^NPAY^195^ or W688A in KRIT1 rescues cortical actin structures while suppressing myosin light chain phosphorylation

**Fig. 8.**
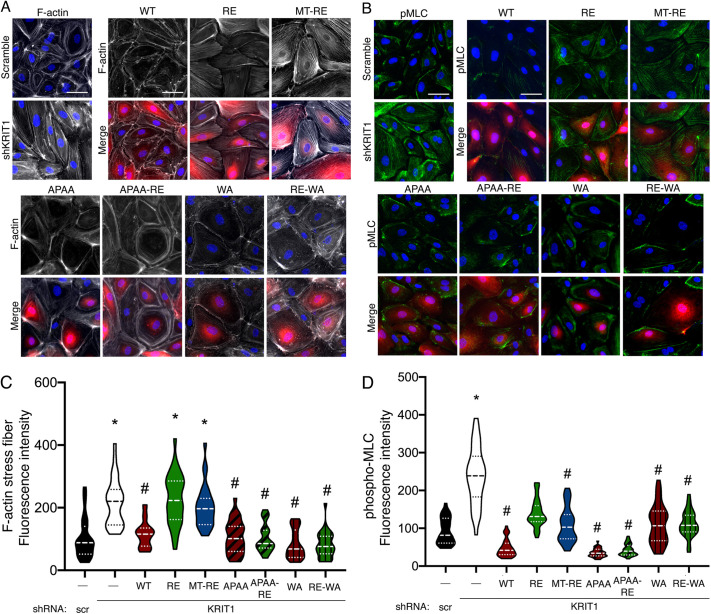
**KRIT1 constructs that rescue barrier function maintain cortical actin structures and suppress MLC phosphorylation.** (A,B) HPAEC were transduced with scramble or KRIT1-targeting shRNA (shKRIT1) alone or in combination with KRIT1 constructs (red), and stained with FITC-phalloidin (A, white) or immunostained for phosphorylated MLC2 (B, green). Nuclei were labeled with Hoechst 33258 and are shown in blue. FITC-phalloidin staining was pseudo-colored white using Adobe Photoshop. Representative epifluorescent images from 15 fields per condition across *n*=4 independent experiments for phalloidin staining (A), or 17–19 fields per condition across *n*=4 independent experiments for pMLC staining (B). Scale bars: 25 µm. (C,D) Quantification of actin stress fiber (C) and pMLC (D) fluorescence intensities were calculated using ImageJ software. Violin plots represent data point frequency distribution between minimum and maximum values, with quartiles indicated by dotted lines, and median indicated by a dashed line. Data shown are from *n*=27–30 cells from five independent experiments for phalloidin staining (C), or *n*=34–38 cells from four independent experiments for pMLC staining (D). For both phalloidin and pMLC analysis, overall *P*<0.0001 by one-way ANOVA; *P<0.01 vs scramble shRNA, #*P*<0.01 vs shKRIT1 by Bonferroni post-hoc testing.

Cell–cell and cell–matrix adhesions are known to strongly influence the actin cytoskeleton, as actin fibers are anchored at these adhesions (Mui et al., 2016; Weber et al., 2011). Additionally, it is known that loss of KRIT1 expression promotes stress fiber formation and RhoA signaling (Glading et al., 2007; Lisowska et al., 2018; Stockton et al., 2010). Given that we observed differences in cell–cell and cell–matrix adhesion between our constructs, we sought to examine whether these changes were accompanied by alteration of the actin cytoskeleton or RhoA signaling [as indirectly measured by myosin light chain (MLC) phosphorylation]. Staining of actin fibers with FITC-phalloidin revealed the formation of stress fibers after knockdown of endogenous KRIT1 (Fig. 8A), concomitant with increased pMLC immunostaining intensity (Fig. 8B). This was consistent with previous reports (Faurobert et al., 2013; Lisowska et al., 2018; Stockton et al., 2010). Overexpression of our constructs after endogenous knockdown revealed that barrier-rescuing constructs (WT, APAA, APAA-RE, WA, RE-WA) promoted the maintenance of cortical actin structures (Fig. 8A), and lower levels of pMLC staining (Fig. 8B). These observations were confirmed by actin stress fiber or pMLC fluorescence intensity quantification (Fig. 8C,D). Interestingly, expression of RE and MT-RE KRIT1 constructs stimulated stress fiber formation but suppressed pMLC levels, though not to the level of barrier-rescuing constructs. This might indicate a minor regulatory divergence between activation of RhoA and the observed changes in the actin cytoskeleton or could be an artifact of overexpression. Regardless, the increase in stress fiber formation in the RE- and MT-RE-expressing cells is consistent with barrier and junctional destabilization and fibrillar adhesion formation.

## DISCUSSION

As a scaffolding protein, KRIT1's physiological function is intimately tied to its binding interactions. Although previous studies have illuminated the nature and molecular mechanism of these interactions, much less is known about how individual binding partners affect critical functional outcomes of KRIT1 signaling, for example, endothelial barrier stability. In the present study, we provide the first systematic exploration of the role of Rap1 and ICAP1α binding in the barrier-promoting function of KRIT1. Using fluorescently labeled KRIT1 constructs, we unveiled that membrane targeting of KRIT1 via lipid modification of KRIT1-RE promoted its enrichment in membrane fractions and localization to cell–cell contacts, similar to WT KRIT1 (Fig. 2), but did not rescue barrier function in the absence of Rap1 binding (Fig. 3). Though it is possible that the C-terminal lipidation disrupted the function of KRIT1, this seemed unlikely given that both membrane-targeted constructs were present in individual confocal slices with β-catenin, suggesting appropriate targeting of these constructs, and that MT-APAA-RE was able to stabilize endothelial AJ and barriers. Additionally, both membrane localization and Rap1 binding appeared dispensable for junctional stabilization in the presence of a mutated ^192^NPAY^195^ motif (APAA, Fig. 5). The APAA mutation reduced ICAP1α binding and expression (Fig. 4) yet restored barrier function as well as WT, suggesting that the ability to bind ICAP1α is also not required. We also introduced a conservative mutation in the KRIT1 PTB domain, concomitant with mutation of the Rap1 binding site, which also stabilized barrier function, though not to the same extent as WT KRIT1 (Fig. 6). In sum, these data strongly suggest that disruption of the intramolecular interaction between the N- and C-termini of KRIT1 is critically important for its ability to stabilize endothelial barriers. Downstream, rescue of endothelial barrier function with KRIT1 constructs containing the APAA or WA mutations correlated with lower levels of β1 integrin activation and smaller, more peripherally localized β1-integrin-containing adhesions (Fig. 7), in addition to maintenance of cortical actin and lower MLC phosphorylation (Fig. 8). Our results suggest that KRIT1-mediated junctional and barrier stabilization is associated with altered β1 integrin activity. Unexpectedly, this can be achieved in the absence of the KRIT1–Rap1 interaction and junctional localization of KRIT1.

Our data challenges the existing paradigm of KRIT1 signaling, which holds that Rap1 binding is critical to the ability of KRIT1 to stabilize endothelial cell–cell contacts because it promotes the junctional localization of KRIT1. However, we observed that expression of APAA-RE and MT-APAA-RE, which bind substantially less Rap1 (Fig. S4), can reverse the disruption of β-catenin localization and the loss of barrier function caused by depletion of endogenous KRIT1 (Fig. 5). Although our Rap1 pulldown reveals that the mutation of Arg^452^ to Glu does not block the interaction with active Rap1 completely, the R452E-containing constructs also bound inactive (GDP bound) Rap1 in our assays, suggesting the presence of a non-specific interaction that may explain the remaining interaction. In contrast, WT KRIT1 shows a strong preference for binding to active Rap1 (Figs S1 and S4). Thus, these data strongly suggest that Rap1 binding is not required for KRIT1 to increase cell junction stability. The ability of APAA-RE KRIT1 expression to rescue AJ stability and barrier function was also unanticipated, as our previous working model considered the junctional localization of KRIT1 to be functionally necessary. However, as is clearly demonstrated by confocal microscopy (Fig. 5), this mutant does not localize to cell–cell contacts. Thus, it appears that enrichment at cell junctions is also not required for KRIT1 to promote endothelial barrier function. Although these data do not eliminate a role for Rap1 and junctional localization in regulating KRIT1 function, they point to the need to adjust our current model of KRIT1 signaling.

Instead, our data indicate a key role for the intramolecular interaction between the N-terminal ^192^NPAY^195^ motif and the C-terminal PTB domain in regulating KRIT1 function. Following our initial observation that MT-RE successfully localized to cell–cell contacts but was unable to rescue β-catenin localization and barrier function, we hypothesized that the loss of Rap1 binding could stabilize this intermolecular interaction, and thus limit the function of KRIT1. Although Béraud-Dufour et al. reported that Rap1 binding did not affect the interaction of N- and C-terminal fragments of KRIT1 (Béraud-Dufour et al., 2007), experiments in our lab suggested that Rap1 binding to full-length KRIT1 protein does affect this interaction (A.J.G., unpublished data). We generated ^192^NPAY^195^ mutants (APAA) that would eliminate this intramolecular interaction to test this hypothesis. However, this approach was complicated by the fact that this site is also critical to the interaction with ICAP1α, a known negative regulator of β1 integrin. Furthermore, as reported previously (Faurobert et al., 2013), loss of KRIT1–ICAP1α binding appears to reduce the stability of ICAP1α protein, which we also observed (Fig. 4). Thus, the APAA mutation abolishes both ICAP1α binding and expression in addition to blocking the N- and C-terminal interaction. However, we noted that although our APAA constructs did exhibit reduced interaction with ICAP1α, the ability to bind and express ICAP1α did not correlate with the ability of our KRIT1 constructs to restore barrier function in KRIT1-depleted cells. As shown in Fig. 5, APAA constructs, which would be predicted to have low ICAP1α expression, and WT KRIT1, which had high ICAP1α expression, rescued barrier function to a similar extent. Next, we identified a mutation in the PTB domain of KRIT1 that did not significantly affect protein folding, and was predicted to disrupt the interaction of the PTB with ^192^NPAY^195^. We introduced this mutation (W688A) into full-length KRIT1 in the presence and absence of the Rap1 binding mutation (RE). Both WA and RE-WA KRIT1 constructs rescued barrier function, limited β1 integrin activity and restored cortical actin, though to a lesser extent than the APAA mutation (Figs 6–8). Together, we believe these data indicate the release of the putative N- and C-terminal interaction as being critical to the ability of KRIT1 to stabilize endothelial cell junctions, as this interaction is ablated in the APAA- or WA-containing constructs, and in WT KRIT1 the intramolecular interaction is subject to regulation by Rap1 binding, etc. Clearly, more work is necessary to test this hypothesis, and we are currently developing the appropriate tools to do so.

Although ICAP1α binding, Rap1 binding, and junctional localization were not required for the ability of KRIT1 expression to stabilize endothelial junctions, we did find that loss of KRIT1 increased β1 integrin activity, and that reversing this increase in integrin activity correlated with increased barrier function in the endothelial monolayer. Loss of KRIT1 has been shown to increase β1 integrin activity (Faurobert et al., 2013; Lisowska et al., 2018; Renz et al., 2015), though others have reported that the loss of KRIT1 promotes the ICAP1α interaction with β1 integrin and subsequently reduces β1 integrin activity (Liu et al., 2013). In the context of this study, cells expressing KRIT1 shRNA exhibited larger, more fibrillar, active β1-integrin-containing adhesions, suggesting an overall increase in β1 integrin activity. Although this increase could be caused by loss of ICAP1α expression, it is worth noting that if the loss of the KRIT1–ICAP1α interaction was suppressing β1 integrin activity via an increase in free ICAP1α, the adhesion pattern in cells expressing APAA constructs would likely be different from the pattern seen in WT and scramble conditions – as the KRIT1-APAA mutation destabilizes ICAP1α protein, thus reducing its abundance (Faurobert et al., 2013, Fig. 4A). If β1 integrin activity was being inhibited by ICAP1α stabilization in scramble and WT conditions, one might expect similar integrin activation levels in the RE and MT-RE conditions as well, but this is not the case. Therefore, it seems unlikely that the alterations in β1 integrin activation we observe can be explained by changes in ICAP1α-mediated β1 integrin regulation.

Nonetheless, it is well established that cell–matrix adhesion and cell–cell adhesion are intimately connected. For example, cell–matrix adhesion has been shown to positively regulate AJ formation (Pulous et al., 2019; Wang et al., 2006; Yamamoto et al., 2015). Indeed, localization of both active β1 and β3 integrins to regions of cell–cell contacts correlates with cadherin organization at cell–cell contacts and AJ stability (Pulous et al., 2019; Sakamoto et al., 2006). However, the degree of integrin activation appears to be important. While in some cases antibody-mediated inhibition of the β1-integrin–matrix interaction results in disruption of cell barriers and increased permeability (Lampugnani et al., 1991; Ojakian et al., 2001; Pulous et al., 2019), under inflammatory conditions (which stimulate integrin activation) subsaturating concentrations of β1-integrin-blocking antibody have been shown to protect endothelial barriers against disruption (Hakanpaa et al., 2015). We noted that in conditions where both adherens junctions and barrier function were stabilized (i.e. scr, rescue with WT, APAA, APAA-RE, WA, RE-WA), we observed active β1 integrin at cell–cell contacts, which supports the idea that integrin activity promotes barrier function (Hakanpaa et al., 2018; Yamamoto et al., 2015; Izawa et al., 2018). Conversely, loss of KRIT1 increased β1 integrin activation, consistent with previous studies (Faurobert et al., 2013; Lisowska et al., 2018), and this integrin phenotype correlated with barrier disruption in KRIT1 shRNA, and RE- and MT-RE-expressing cells. Taken together, these results imply that regulation of both cell–cell and cell–matrix adhesions is necessary for KRIT1-mediated barrier stabilization.

This suggests a model whereby KRIT1 regulates the location and magnitude of β1 integrin activation and AJ stability. However, it is less clear in which order these events occur. How this mechanism fits into the context of aberrant KRIT1 signaling in CCM is also unclear. Several studies have linked increased β1 integrin activity downstream of loss of CCM proteins with upregulation of MEKK3 (also known as MAP3K3) and KLF2 and KLF4, and/or RhoA signaling (Faurobert et al., 2013; Lisowska et al., 2018; Macek Jilkova et al., 2014; Renz et al., 2015), both of which are associated with perturbed barrier function. MEKK3 binds to CCM2; this interaction limits the ability of MEKK3 signaling to increase expression of the transcription factors KLF2 and KLF4 (Cuttano et al., 2016; Hong et al., 2020; Lopez-Ramirez et al., 2019; Renz et al., 2015; Zhou et al., 2015, 2016), which in turn promote CCM lesion formation by altering gene expression (Hong et al., 2020). In this study, we show that all of our APAA KRIT1 constructs bind to CCM2 in an equivalent manner (Fig. 4). Furthermore, whereas *Klf2* mRNA expression was increased in KRIT1-shRNA-expressing cells, expression of RE and APAA constructs rescued *Klf2* expression to similar levels, despite having opposing effects on barrier function (Fig. S5). Although upregulation of KLF2 has been linked to increased β1 integrin activity after loss of CCM proteins (Renz et al., 2015), our data suggests that altered MEKK3-KLF2/4 signaling does not explain the effect of KRIT1-mediated β1 integrin activity on the endothelial barrier. One possible reason for this discrepancy is differences in the role of KLFs between arterial endothelial cells and microvascular endothelial cells. In addition, the effects of KLF2 in the context of CCM have primarily been studied in whole animal models, with observations of lesion development and vascular morphological defects (Hong et al., 2020; Renz et al., 2015; Zhou et al., 2015, 2016). While our observations suggest that KLF2 signaling does not mediate the barrier phenotype in HPAEC, further work will be necessary to examine this mechanism in endothelial cells of varying origin.

On the other hand, we and others have also established the presence of a strong pro-inflammatory phenotype in CCM lesions, which appears to involve an increase in reactive oxygen species (ROS) (Antognelli et al., 2018; Marchi et al., 2015; Retta and Glading, 2016), VEGF-A (DiStefano and Glading, 2020; DiStefano et al., 2014; Kar, 2015) and NF-κB activation (Goitre et al., 2017). As β1 integrin activation and the formation of fibrillar adhesions are involved in the endothelial inflammatory response (Al-Yafeai et al., 2018; Hakanpaa et al., 2015), it is also possible that the barrier rescue we observe in KRIT1 APAA mutants is associated with a downregulation of inflammatory signaling. Future studies are needed to explore this hypothesis, and whether this is coupled to a change in the conformation of KRIT1. Clearly, additional studies are needed to fully understand how KRIT1 affects the complex role that cell–matrix interactions play in regulating AJ formation and endothelial barrier stability. Understanding these pathways could provide insight into the mechanisms and efficacies of new pharmacologic therapies for treating CCM.

## MATERIALS AND METHODS

### Cell culture

Human pulmonary artery endothelial cells (HPAEC; Cell Applications, Inc., San Diego, CA, Lot #2228) were cultured in Dulbecco's Modified Eagle's Medium DMEM/F-12 (1:1 ratio), supplemented with 5% fetal bovine serum (FBS), 1% antibiotic-antimycotic solution (Gibco/Thermo Scientific, Waltham, MA), 1% endothelial cell growth supplement (ECGS; ScienCell, Carlsbad, CA), and 50 µM heparin (Calbiochem, La Jolla, CA). Only cell passages 2–5 were used for experiments. HEK293A (Invitrogen, Carlsbad, CA) and HEK293T cells (a gift from Gregory Tall, University of Michigan) were cultured in DMEM containing high glucose, supplemented with 5% FBS, 1% penicillin/streptomycin/L-glutamine (Gibco/Thermo Scientific), and 1% nonessential amino acids (Gibco/Thermo Scientific). HEK293T cells used to produce lentiviral particles were cultured in DMEM containing high glucose supplemented with 10% FBS, 1% Glutamax (Gibco/Thermo Scientific), and 1% nonessential amino acids (Gibco/Thermo Scientific), with no antibiotic. All cells were cultured at 37°C with 5% CO_2_.

### Plasmids, mutagenesis and transfections

KRIT1 adenoviral constructs were generated by cloning mCherry-tagged KRIT1 (WT) into the adenoviral shuttle vector pDC315 (De Luca et al., 2020), which was co-transfected with the adenoviral parent plasmid pBHGloxΔE1,3Cre (both gifts from Alan Smrcka, University of Michigan) to produce adenovirus. pMDLg/pRRE, pRSV/Rev and pMD2.G plasmids were used to produce lentiviral particles. pMDLg/pRRE, pRSV-Rev and pMD2.G were deposited by Didier Trono (Addgene plasmid #12251, http://n2t.net/addgene:12251, RRID: Addgene_12251; Addgene plasmid #12253, http://n2t.net/addgene:12253, RRID: Addgene_12253; Addgene plasmid #12259, http://n2t.net/addgene:12259, RRID:Addgene_12259) (Dull et al., 1998). pLKO.1-scramble shRNA was deposited by David Sabatini (Addgene plasmid #1864, http://n2t.net/addgene:1864, RRID: Addgene_1864) (Sarbassov et al., 2005). KRIT1-targeting shRNA *TRCN0000072879* cloned into the pLKO.1 vector was purchased from The RNAi Consortium (Open BioSystems, Huntsville, AL). pGEX-2T plasmid was a gift from Denise Hocking (University of Rochester), and pGEX2TK-F123, pGEX-Rap1a-WT, pcDNA3.1-CCM2-Myc/His and pEF4-ICAP1α-Myc/His plasmids were gifts from Mark Ginsberg (University of California, San Diego).

Mutation of R452E (to generate RE-KRIT1) and insertion of the Rap1 polybasic region and lipid modification signal (KKKPKKKSCLLL) or the negative lipidation control sequence (KKKPKKKSSLLL) at the C-terminus of KRIT1 to generate MT-RE or SL constructs (respectively) was accomplished using a QuikChange II site-directed mutagenesis kit (Agilent, Santa Clara, CA). Mismatch mutations conferring resistance to KRIT1-targeting shRNAs were also generated by site-directed mutagenesis in all KRIT1 constructs. An In-Fusion HD cloning kit (Takara Bio, Kusatsu, Shiga, Japan) was subsequently used to introduce the ^192^NPAY^195^ to ^192^APAA^195^ double mutation (APAA, APAA-RE, MT-APAA-RE), the W688A mutation (WA, RE-WA), and to generate MT-RE and MT-APAA-RE constructs containing a stop codon after the C-terminal cysteine of the lipid modification sequence (KKKPKKKSC) for exclusive use in the Rap1 pulldown experiments to reduce non-specific binding.

### Adenovirus and lentivirus production

To produce adenovirus containing each of our KRIT1 constructs, HEK293A cells were transfected with parent plasmid pBHGloxΔE1,3Cre and shuttle vector pDC315 containing the gene of interest using TurboFect transfection reagent (Thermo Scientific). Harvested viral particles were desalted using a Sephadex G-25 PD-10 Desalting Column (GE Life Sciences, Marlborough, MA) and collected in 10 mM Tris, pH 8.0, containing 10% glycerol. Viral titers were determined by immunoreactivity ‘spot’ assay, as previously described (Bewig and Schmidt, 2000), and KRIT1 constructs were transduced into HPAEC at appropriate multiplicities of infection (MOI) to achieve equal expression levels between constructs.

Lentiviral particles were produced by transfecting HEK293T cells with packaging plasmid pMDLg/pRRE, envelope plasmid pMD2.G, transcription factor plasmid pRSV-Rev, and transfer vector pLKO.1 containing either scramble shRNA or KRIT1 shRNA *TRCN0000072879*, using Lipofectamine 2000 (Invitrogen). Lentiviral particles were precipitated, and then collected in serum-free DMEM/F-12 (1:1). HPAEC were transduced with lentivirus in complete medium containing 8 µg/ml hexadimethrine bromide (Polybrene; Sigma-Aldrich, St Louis, MO).

### Antibodies

For western blotting, polyclonal rabbit anti-KRIT1 (Abcam, Cambridge, UK) was used at a dilution of 1:1000, and mouse monoclonal anti-tubulin-α (Clone DM1a; Neomarkers, Inc., Portsmouth, NH) was used at a 1:500 dilution. For subcellular fractionation experiments, polyclonal rabbit anti-Na^+^/K^+^-ATPase α1 subunit, polyclonal rabbit anti-RhoGDI and monoclonal rabbit anti-histone H3 (Clone D1H2) were purchased from Cell Signaling Technology (CST; Danvers, MA); all primary antibodies for subcellular fractionation western blots were used at a dilution of 1:500. Mouse anti-mCherry antibody (Takara Bio) was used for immunoprecipitation; ICAP1α-Myc/His and CCM2-Myc/His were detected using monoclonal rabbit anti-Myc tag (Clone 71D10; CST) at a 1:1000 dilution. Horseradish peroxidase (HRP)-linked horse anti-mouse IgG or donkey anti-rabbit IgG secondary antibodies were purchased from CST and used at a dilution of 1:2000.

For immunofluorescence experiments, monoclonal mouse anti-β-catenin (Clone 6F9, Sigma-Aldrich, 1:5000 dilution) or rabbit anti-pMLC2 (Thr18/Ser18, CST, 1:500 dilution) was used, followed by AlexaFluor 488-conjugated goat anti-mouse or anti-rabbit IgG secondary antibodies (Life Technologies/Invitrogen, 1:1000 dilution). Staining of activated β1 integrin was performed using an AlexaFluor 488-conjugated anti-active β1 integrin antibody (Clone HUTS4, EMD Millipore) at a 1:500 dilution. For immunostaining in adenoviral titer assays, goat anti-hexon primary antibody (Meridian Life Sciences, Memphis, TN) was used at a 1:1000 dilution, and HRP-linked rabbit anti-goat IgG secondary antibody (Invitrogen) was used at a 1:500 dilution.

### Western blotting

Lysates were prepared by washing cells with 1× Tris-buffered saline (TBS; pH 7.4), then scraping cells into lysis buffer (50 mM Tris pH 7.4, 150 mM NaCl, 5 mM MgCl_2_, 1% Triton X-100) containing cOmplete mini EDTA-free protease inhibitor tablet (Roche, Basel, Switzerland) and phosphatase inhibitor cocktail (PPI; final concentration 100 µM Na_4_P_2_O_7_, 1 mM NaF, 1 mM Na_3_VO_4_, 300 µM β-glycerophosphate, 2 mM imidazole, 4 mM sodium tartrate). Lysates were incubated at 4°C for 20 min with constant agitation, then clarified by centrifugation at 18,000 ***g*** for 10 min. Protein content was quantified using a bicinchoninic acid (BCA) assay kit (Pierce/Thermo Scientific) according to the manufacturer's instructions. Appropriate volumes of lysate were mixed with Laemmli sample buffer and heated at 95°C for 10 min. Samples were resolved by SDS-PAGE on precast 4–20% Tris-HCl gels (BioRad, Hercules, CA), transferred to a 0.45-µm nitrocellulose membrane, then blocked in 5% (w/v) nonfat dry milk (NFDM) in 1× TBS plus 0.1% Tween 20 (TBST). Primary antibody was diluted in TBST plus 5% NFDM, and the membrane was incubated in primary antibody overnight at 4°C while rocking. The membrane was washed three times for 5 min with TBST and incubated in secondary antibody (diluted in 5% NFDM/TBST) for 1 h at room temperature. The membrane was then washed three times for 5 min with TBST to remove excess secondary antibody. Next, either SuperSignal West Femto or Pico enhanced chemiluminescent substrate (Thermo Scientific) was applied to the membrane, which was then imaged on a BioRad ChemiDoc imaging system. Densitometry quantification was performed using Image Lab 6 software (BioRad). When blotting for multiple proteins was necessary, blots were cut and probed separately. In some cases, blots were stripped with a mild stripping buffer [1.5% (w/v) glycine, 0.1% SDS, 1% Tween 20, pH 2.0–2.2] before incubation with another set of primary and secondary antibodies.

### GST-Rap1 purification and KRIT1 pulldown

BL21(DE3) *E. coli* cells (Lucigen, Middleton, WI) transformed with either pGEX-2T (GST only) or pGEX-Rap1a (GST-Rap1a) were shaken at 225 rpm in a 37°C incubator until the OD_600_ reached 0.6. Protein expression was then induced by addition of 0.1 mM (GST) or 0.2 mM (GST-Rap1a) isopropyl β-D-1-thiogalactopyranoside (IPTG; Gold Biotechnology, St Louis, MO), followed by additional incubation for 3 h at 37°C or 18 h at 16°C, respectively. Bacteria expressing the recombinant proteins were pelleted and resuspended in purification buffer (50 mM Tris, pH 7.4, 150 mM NaCl, 5 mM MgCl_2_) containing 1 mg/ml lysozyme (Gold Biotechnology), protease inhibitor tablet (Roche) and PPI. Guanosine diphosphate (GDP; Sigma-Aldrich) was added at 100 µM to lysates containing GST-Rap1a to stabilize the protein structure. Lysates were agitated gently for 20 min at 4°C, then lysed by sonication on ice using a Branson Sonifier 250 (four 15-s pulses at 20% output, Branson Ultrasonics, Danbury, CT). Lysates were then clarified by centrifugation. The supernatants were added to GSH-Sepharose beads (GE Life Sciences) equilibrated in purification buffer and incubated at 4°C for 2 h with agitation. Beads containing immobilized protein were washed three times with purification buffer then immediately used for pulldown experiments.

Forty-eight hours prior to each pulldown experiment, HEK293A cells were transfected with the appropriate mCherry-KRIT1 constructs using Lipofectamine 2000. Immediately prior to the pulldown experiment, immobilized GST-Rap1a protein was loaded with guanine nucleotide by incubation with 10 mM GDP or 1 mM guanosine [γ-thio]triphosphate (GTPγS; Sigma-Aldrich) in loading buffer (50 mM Tris, pH 7.4, 150 mM NaCl, 5 mM MgCl_2_, 10 mM EDTA) for 45 min at 30°C. Samples were mixed by inverting multiple times every 5 min. After the 45-min incubation, MgCl_2_ was added to a final concentration of 65 mM and samples were incubated on ice for 30 min while lysates were prepared. Cells were lysed and processed as described above for western blotting. Three hundred µg of total cell protein was added to individual tubes containing 10 µg of immobilized recombinant GST, GST-Rap1a protein loaded with GDP, or GST-Rap1a protein loaded with GTPγS, in the presence of 5 mM dithiothreitol (DTT). Samples were incubated at 4°C overnight with constant agitation to maximize binding. Subsequently, samples were washed three times for 5 min in lysis buffer containing 250 mM NaCl and 5 mM DTT, then resuspended in Laemmli sample buffer and heated at 95°C for 10 min. Samples were western blotted and imaged for band density quantification as described above. Results were analyzed by two-way ANOVA with Tukey post-hoc testing. To detect equal loading of recombinant proteins, nitrocellulose membranes were stained with 0.1% (w/v) Ponceau S (Sigma-Aldrich) in 1% (v/v) glacial acetic acid.

### Subcellular fractionation

HEK293A cells transfected with mCherry-KRIT1 constructs were fractionated into subcellular components by differential centrifugation. Cells were scraped off of tissue culture plates into 1× phosphate-buffered saline (PBS) containing protease inhibitor tablet (Roche) and PPI, then pelleted. The resulting pellets were resuspended in fractionation buffer (20 mM Tris, pH 7.4, 100 mM sucrose, protease inhibitor, PPI) and homogenized with eight passes of a Teflon pestle in a Dounce homogenizer. Ten percent of the total lysate volume was saved as the total fraction (whole cell lysate). The remaining lysate was then centrifuged at 2000 ***g*** for 10 min to remove the nuclei. The resulting supernatant was collected, and the spin was repeated to ensure removal of all nuclei. Next, the supernatant was centrifuged for 30 min at 18,000 ***g*** to pellet the plasma membrane. The supernatant from this third spin was collected and saved as the cytosolic fraction. The plasma membrane pellet was then washed with fractionation buffer and the spin was repeated. Proteins in the total and plasma membrane fractions were solubilized with 1% Triton X-100 and 0.1% (w/v) SDS (final concentrations) at 4°C for 20 min with constant agitation prior to measurement of protein content (BCA, Pierce/Thermo Scientific). Equal protein amounts for each fraction were then mixed with Laemmli sample buffer, heated at 95°C for 10 min, and western blotted. Blots were processed as described above, and results were analyzed using one-way ANOVA with Tukey post-hoc testing.

### Immunofluorescence and image analysis

Glass coverslips (#1.5 thickness, Warner Instruments, Hamden, CT) were flame sterilized and coated with 10 µg/ml human fibronectin (FN; isolated from expired human plasma, URMC Blood Bank, Rochester, NY) at 37°C. HPAEC were seeded on coverslips at 80% confluence and transduced with lentivirus. After 24 h, the lentivirus was removed, and cells were transduced with adenoviral KRIT1 constructs as indicated. The cells were then allowed to grow to 100% confluence. The confluent monolayer was then washed with 1× PBS, fixed in 10% formalin for 20 min, permeabilized in 0.2% Triton X-100 for 5 min, and blocked for 1 h in 10% normal goat serum. Samples were then incubated in either mouse anti-β-catenin (1:5000, Sigma-Aldrich) or rabbit anti-pMLC2 (1:500) followed by AlexaFluor 488-conjugated anti-mouse or anti-rabbit IgG (1:1000, Invitrogen) for 1 h. For activated β1 integrin staining, cells were incubated with AlexaFluor 488-conjugated mouse anti-β1 integrin (Clone HUTS4, 1:500, EMD Millipore). For phalloidin staining, samples were incubated with 330 nM FITC-phalloidin (Alexis Biochemical, San Diego, CA) for 1 h. Coverslips were washed three times with 0.001% Triton X-100 after each antibody incubation. All samples were counterstained with 1 µg/ml Hoechst 33258 stain (Invitrogen) to label nuclei, then mounted on glass slides in ProLong Gold Antifade mounting medium (Invitrogen).

Confocal imaging was performed on a Nikon A1R HD laser scanning confocal microscope with a 60× (n.a. 1.49) oil objective using NIS-Elements C software (Nikon, Tokyo, Japan). Images were processed in Adobe Photoshop (Adobe, San Jose, CA). β-catenin staining coverage and junctional continuity were analyzed using Junctional Analysis Program [JAnaP (Gray et al., 2019); Stroka Lab, University of Maryland]. Briefly, cell perimeters were way-pointed based on β-catenin staining intensity, with two or three cells analyzed per field. A pixel intensity threshold of 15 (on a 0–255 scale) was set to limit background. Pixels above this threshold were considered positively stained and used to calculate the extent of staining coverage and continuity at the cell perimeter. Any segment of positive staining that was 15 px or longer was considered continuous. Based on the pixel ratio of these images (288 nm/px), continuous segments were determined to be 4.3 µm or longer. Both staining coverage and continuity were reported as percentages of the cell perimeter. The width of β-catenin junctional staining was assessed by line scan using ImageJ software. Images were background corrected, and then a 15-µm line segment was drawn perpendicular to the edge of a cell at the midpoint of the cell edge. To eliminate the contribution of diffuse staining seen at destabilized junctions, positively stained junctional regions were considered to be greater than 5000 arbitrary fluorescence units (AFU), and junctional width was calculated by determining the length of the line segment with fluorescence intensity above this threshold. Calculations were done using a custom R script. In order to accurately assess average junctional width per cell, three or four measurements were taken for each cell. Results were analyzed using one-way ANOVA with Tukey post-hoc testing.

Active β1 integrin staining was analyzed with Imaris analysis software (Bitplane, Zürich, Switzerland) using the Surfaces module. A region of interest was drawn around each cell that was analyzed and a threshold filter of 5.4 was applied to each image to identify positively stained objects above background signal. The program reported the area, length and width of objects in each cell. A custom R script was created to calculate length:width ratios of each object and determine the median ratio per cell (https://www.R-project.org/). Measurements generated in R were analyzed in GraphPad Prism Software (GraphPad Software Inc., La Jolla, CA) using one-way ANOVA with Tukey post-hoc testing.

Epifluorescence imaging of phalloidin and pMLC staining was carried out using an ORCA-ER digital camera (Hamamatsu, Hamamatsu City, Japan) on an Olympus IX70 microscope (Tokyo, Japan) with an Olympus UPLSAPO 20X objective (n.a. 0.75) using MetaMorph software (Molecular Devices, Sunnyvale, CA). Images were background corrected and analyzed using ImageJ software. For phalloidin line scan analysis, a line perpendicular to the longest axis of the cell was drawn through the cell center, and a custom R script was used to calculate the area under the curve of the histogram generated by the line scan. This value was then divided by the length of the line to yield an average intensity value. For pMLC quantification, cells of interest were outlined manually and the mean fluorescence intensity was measured for each cell. Results were analyzed using one-way ANOVA with Tukey's post-hoc testing.

### Permeability assays

HPAEC transduced with the appropriate lentiviral shRNA were seeded on to Transwell inserts (3 µm pore size, 2×10^6^ pores/cm^2^, Corning Life Sciences, Corning, NY) coated with 10 µg/ml FN. The following day, lentivirus was removed and cells were transduced with the appropriate adenoviral constructs for 24 h. To verify that the cells reached confluence on the opaque membrane, HPAEC were seeded in parallel on FN-coated wells in a 24-well dish and also transduced. Given that the monitor wells demonstrated a confluent monolayer, 20 µg/ml FITC-conjugated dextran (40 kDa; Sigma-Aldrich) diluted in assay medium (0.5% FBS, 0.1% ECGS, without Phenol Red) was then added to the upper chamber of the Transwell and allowed to diffuse through the cell layer for 2 h at 37°C. Medium from the bottom chamber was then collected and loaded on a black 96-well plate (Corning Life Sciences) in triplicate. FITC fluorescence was measured at excitation 485 nm/emission 520 nm using a Synergy H4 hybrid plate reader (BioTek, Winooski, VT) and compared to a standard curve of FITC-dextran diluted in assay medium.

Transendothelial electrical resistance (TEER) measurements were performed using STX3 electrodes on an Epithelial Volt/Ohm Meter (EVOM2; World Precision Instruments), as per the manufacturer's protocol. Briefly, electrodes were equilibrated in assay medium, and then placed in Transwell culture plates containing the same medium mix. Resistance of an FN-coated, cell-free well was measured and subtracted from cell-containing wells as background. The resistance measurements were multiplied by the cell growth area to yield Ω*cm^2^ values. For both dextran permeability and TEER assays, data were analyzed using one-way ANOVA with Tukey post-hoc testing.

### Co-immunoprecipitation

To assess interaction of our KRIT1 constructs with CCM2, we co-transfected each pDC315-mCherry-KRIT1 construct with pcDNA3.1-CCM2-Myc/His in HEK293A cells. As a control, one condition contained empty pDC315 vector in place of mCherry-KRIT1. Cells were lysed and processed for western blotting as described above. Three hundred µg of total cell protein was added to Protein G agarose beads (Gold Biotechnology) equilibrated with lysis buffer, along with 2 µg of mouse anti-mCherry antibody (Takara Bio) or non-immune mouse IgG (isotype control, Santa Cruz Biotechnology, Santa Cruz, CA), and incubated overnight at 4°C. The next day, beads were washed three times with lysis buffer then resuspended in Laemmli buffer, heated at 95°C for 10 min, and western blotted. CCM2-Myc band density was normalized to respective immunoprecipitated mCherry-KRIT1 bands. Results were analyzed using one-way ANOVA with Tukey post-hoc testing.

To assess KRIT1–ICAP1α interaction, we also co-transfected each pDC315-mCherry-KRIT1 construct with pEF4-ICAP1α-Myc/His. Samples were processed as described above, with some modifications. Five hundred µg of total cell protein was used for immunoprecipitation. Additionally, immunoprecipitation samples were pre-cleared with equilibrated Protein G agarose beads (Gold Biotechnology) and 1 µg of non-immune mouse IgG for 1 h at 4°C to reduce background. Pre-cleared lysates were then applied to freshly equilibrated beads with 2 µg of mouse anti-mCherry antibody (Takara Bio) or mouse IgG Isotype control. Samples were then processed as described for co-immunoprecipitation of CCM2.

### RNA isolation and semi-quantitative reverse transcription-PCR (RT-qPCR)

Cells were lysed in TRIzol reagent (Invitrogen) and RNA was isolated using a Direct-zol RNA prep kit (Zymo Research, Irvine, CA) as per the manufacturer's protocol. RNA was eluted in ultrapure H_2_O and 1.5 µg of isolated RNA was used to prepare cDNA using a LunaScript reverse transcriptase kit (New England BioLabs, Inc., Ipswich, MA) as per the manufacturer's protocols. cDNA was then mixed with iTaq Universal SYBR Green Supermix (BioRad) and primer sets targeting either *klf2* (FP: 5′-ACCTACACCAAGAGTTCGCA-3′; RP: 5′-GAAGGCACGATCGCACAGAT-3′) or *Gapdh* (FP: 5′-TGGTATCGTGCAAGGACTCATGAC-3′; RP: 5′-ATGCCAGTGAGCTTCCCGTTAAGC-3′). Amplicons were generated by running samples in triplicate on an ABI PRISM 7000 Sequence Detection System. Expression was calculated using the ΔΔCt method (Pfaffl, 2001) relative to negative control (i.e. scramble shRNA) and normalized to *Gapdh* as an internal control.

### KRIT1-FERM purification and circular dichroism

KRIT1-F123, with or without PTB domain mutations was isolated from bacterial cultures as previously reported (Liu et al., 2011). Briefly, bacterial supernatants were added to a GSH-Sepharose column (GE Life Sciences) and equilibrated in FERM purification buffer (1× PBS, pH 7.2, containing 1% Triton-X 100, 1 mg/ml lysozyme (Gold Biotechnology), bacterial protease inhibitor (Gold Biotechnology) and PPI. The column was washed before removal of GST tag by on-column cleavage using biotinylated thrombin (EMD Millipore) in 20 mM Tris-HCl, 150 mM NaCl, 20 mM n-octyl-β-D-glucoside, pH 7.2. Thrombin was removed by incubating the cleaved protein solution with streptavidin agarose beads (Gold Biotechnology) for 30 min at 4°C while rocking. The protein was then dialyzed against CD buffer (20 mM sodium phosphate, pH 7.2, 150 mM NaF, 20 mM n-octyl-β-D-glucoside) at 4°C, twice for 4 h then overnight, in a Slide-A-Lyzer dialysis cassette (Thermo Scientific).

Circular dichroism (CD) scans were run on a Jasco J-1100 CD Spectrometer (JASCO Inc., Tokyo, Japan) in a quartz cuvette with 1 mm pathlength (JASCO Inc.). Samples were scanned from 260 to 190 nm, with buffer scans used for background subtraction. Two accumulations were taken for each scan. To analyze CD scans, data were converted to molar ellipticity using the measured concentrations of each sample, and were analyzed using DichroWeb (Miles et al., 2021). In order to accurately estimate secondary structure content of the purified proteins, various combinations of analysis programs and reference data sets were run in order to find the parameters in which estimated WT FERM domain secondary structure reflected secondary structures previously determined by X-ray crystallography (Gingras et al., 2012, 2013; Li et al., 2012; Lopez-Ramirez et al., 2021). Once these parameters were identified (program CDSSTR coupled with reference set 4, Sreerama and Woody, 2000), they were applied to all CD samples.

### Statistical analysis

Statistical analyses were performed using GraphPad Prism software (GraphPad Software Inc.), with significance set at α=0.5. Assays with two conditions (Fig. S2A) were analyzed using paired Student's *t*-test; assays with more than two conditions were analyzed using either one-way or two-way ANOVA, and appropriate post-hoc testing, as indicated in the figure legends.

## Supplementary Material

Supplementary information

Reviewer comments
