# Supplementary material for: Contribution of protein–protein interactions to the endothelial-barrier-stabilizing function of KRIT1
Source: J Cell Sci. 2022 Jan 25;135(2):jcs258816. doi: 10.1242/jcs.258816 (PMC8917353; doi:10.1242/jcs.258816)
Supplement: Supplementary information [file joces-135-258816-s1.pdf]

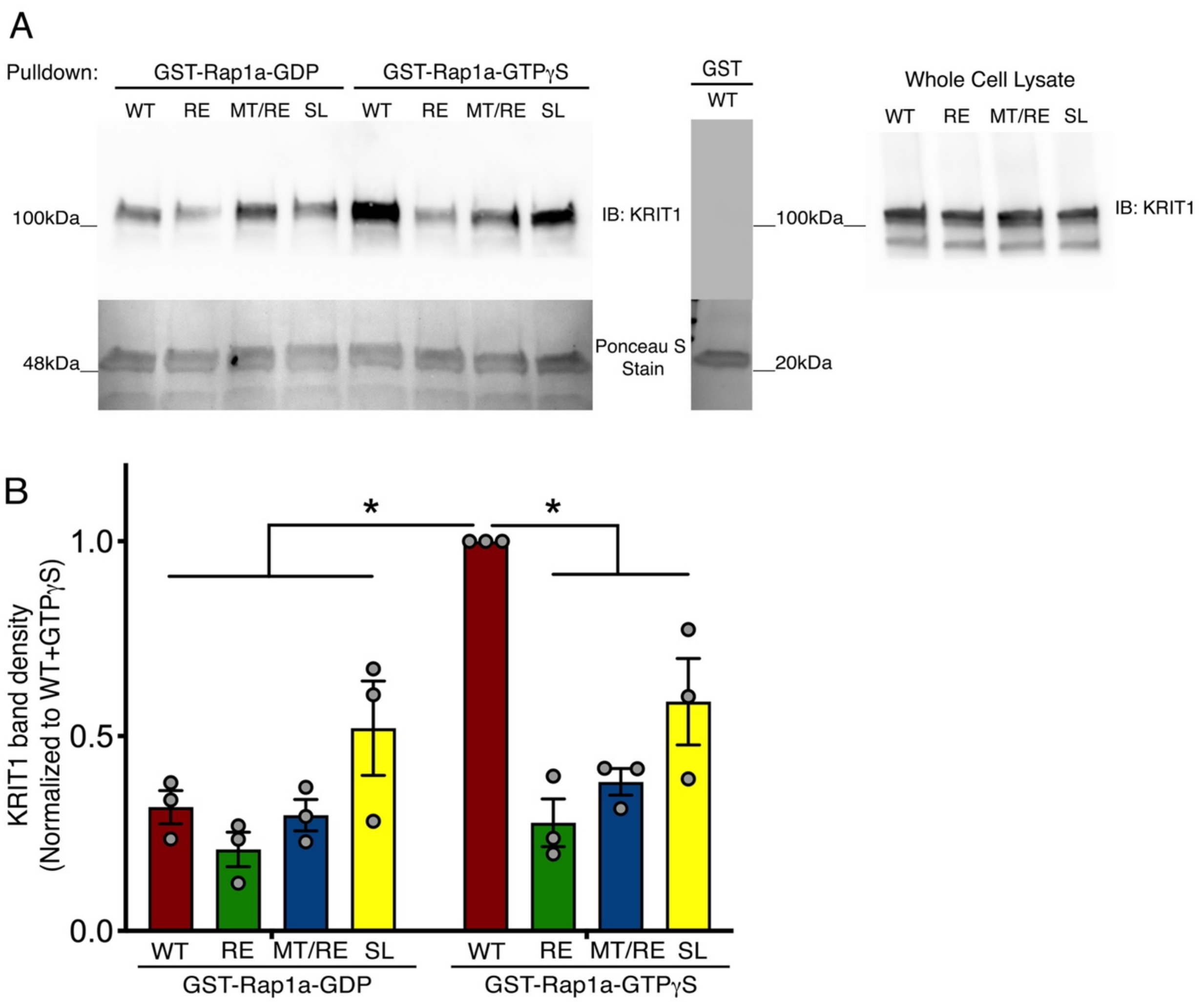

Fig. S1. R452E mutation inhibits KRIT1-Rap1 binding. Lysate from HEK293 cells transfected with KRIT1 constructs was applied to 10ug immobilized recombinant GST-Rap1a(WT) loaded with either GDP or GTPγS. Immobilized GST was also used as a binding control. Pulldowns were Western blotted for KRIT1. Recombinant GST and GST-Rap1a was detected by Ponceau S stain to verify equal loading. Representative blots and densitometry quantification are from n=3 independent experiments. Data shown are mean normalized band densities +/- SEM, normalized to WT KRIT1+Rap1-GTPγS. *$p<0.01$ by Tukey's post-hoc testing vs. WT+GTPγS. $p=0.0014$ by two-way ANOVA.

Journal of Cell Science • Supplementary information

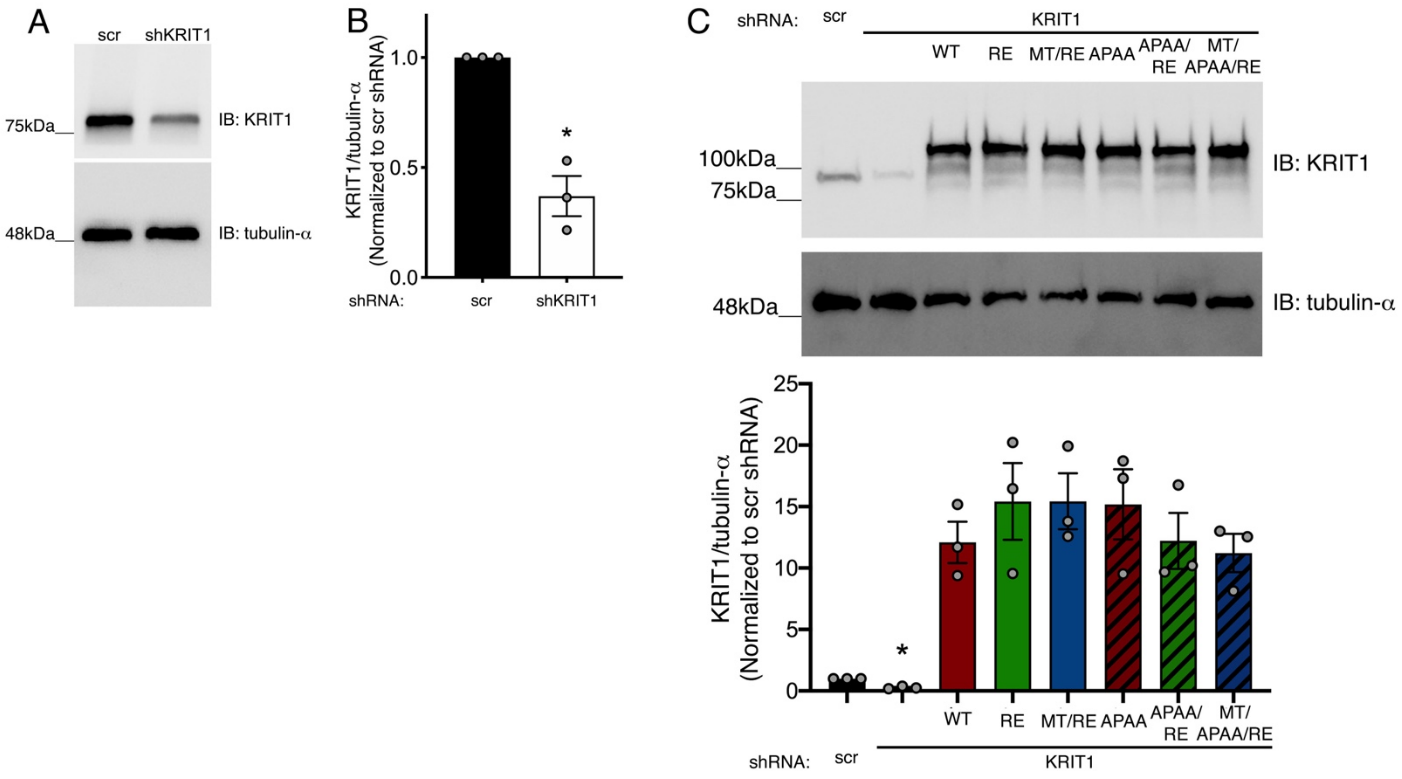

Fig. S2. KRIT1-targeted shRNA efficiently knocks down endogenous KRIT1 and adenoviral constructs overexpress mCherry-KRIT1. (A) Western blot and quantification of endogenous KRIT1 in human pulmonary artery endothelial cells (HPAEC) after transduction with either negative control shRNA (scramble) or KRIT1-targeted shRNA (shKRIT1). Tubulin-α blot is shown as a loading control. **(B)** KRIT1 band density was calculated relative to tubulin-α and normalized to scramble shRNA. Representative Western blots and quantification are from n=3 independent experiments. Data shown are mean +/- SEM. *p<0.05 by paired t-test. **(C)** Overexpression of mCherry-tagged KRIT1 constructs in HPAEC after depletion of endogenous KRIT1. High adenoviral transduction yielded ~12-15x expression of exogenous KRIT1 relative to scramble (endogenous) control. Top KRIT1 bands correspond to mCherry-tagged constructs, and bottom bands are endogenous KRIT1. mCherry-KRIT1 band density was calculated relative to tubulin-α loading control and normalized to scramble shRNA. Representative Western blots and quantification are from n=3 independent experiments. Data shown are mean normalized band densities +/- SEM. *p<0.05 by Tukey's post-hoc testing vs. scramble shRNA alone. p=0.0210 by one-way ANOVA.

Journal of Cell Science • Supplementary information

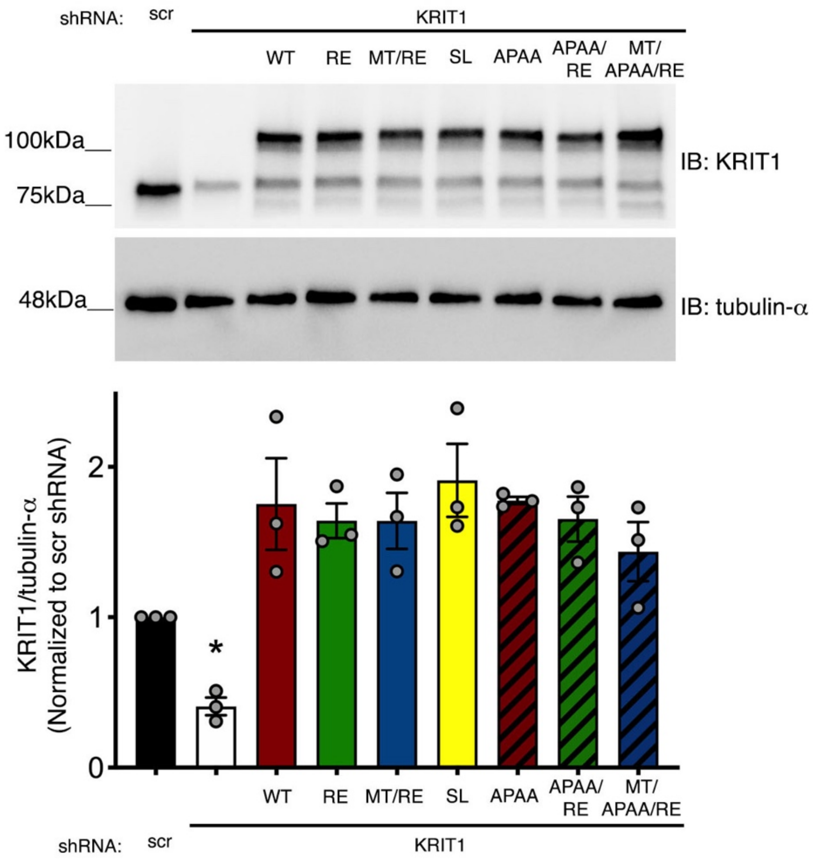

Fig. S3. KRIT1 adenoviral constructs express at roughly endogenous levels in HPAEC. Expression of mCherry-tagged KRIT1 constructs in HPAEC after depletion of endogenous KRIT1. Adenoviral transduction yielded ~1.5x expression of exogenous KRIT1 relative to scramble (endogenous) control. Top KRIT1 bands correspond to mCherry-tagged constructs, and bottom bands are endogenous KRIT1. mCherry-KRIT1 band density was calculated relative to tubulin-α loading control and normalized to scramble shRNA. Representative Western blots and quantification are from n=3 independent experiments. Data shown are mean normalized band densities +/- SEM. *$p<0.05$ by Tukey's post-hoc testing vs. scramble shRNA alone. $p=0.0930$ by one-way ANOVA.

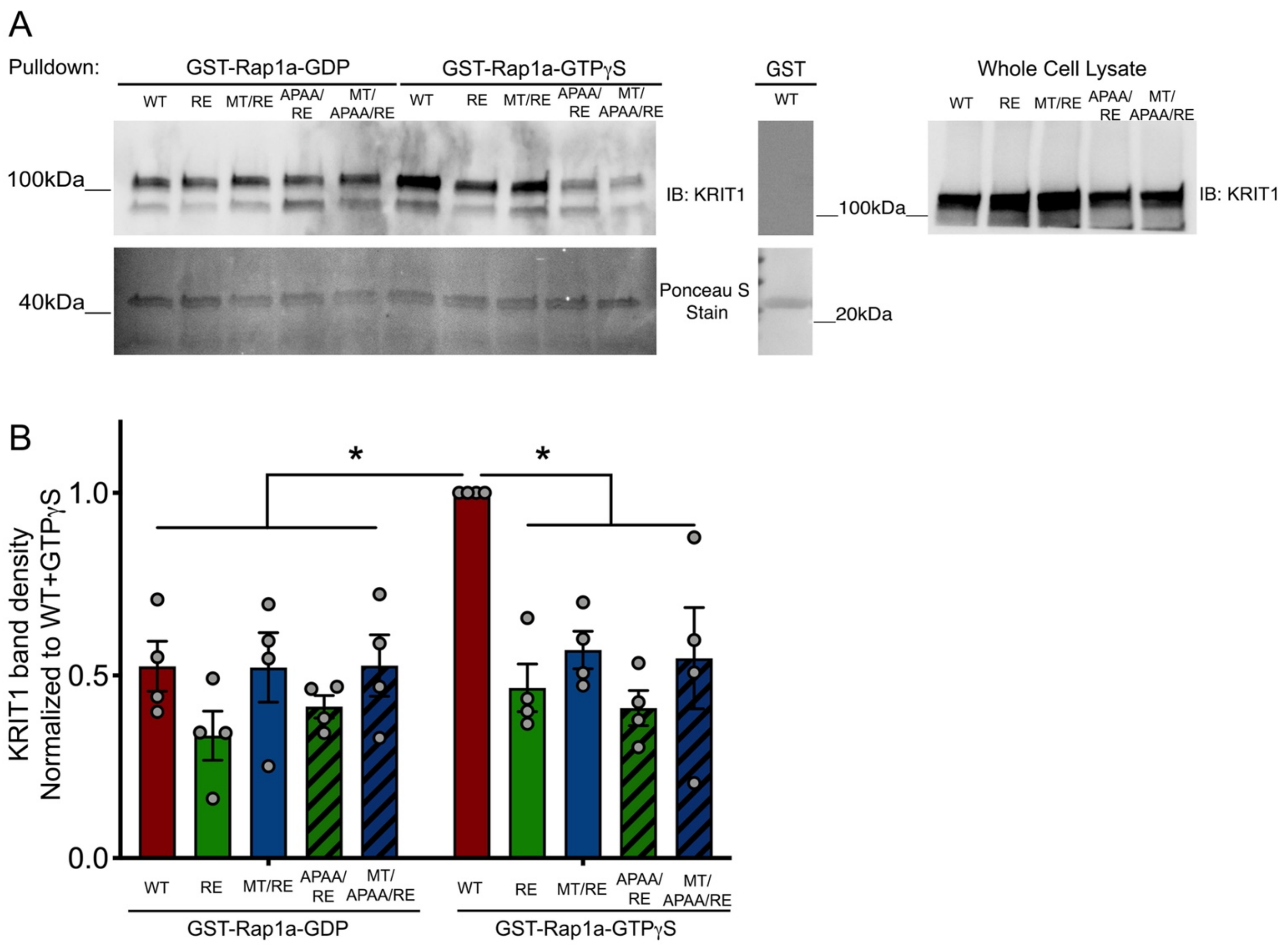

Fig. S4. R452E mutation inhibits KRIT1-Rap1 binding in APAA constructs. Lysate from HEK293 cells transfected with KRIT1 constructs was applied to 10ug immobilized recombinant GST-Rap1a(WT) loaded with either GDP or GTPγS. Immobilized GST was also used as a binding control. Pulldowns were Western blotted for KRIT1. Top KRIT1 bands for pulldown correspond to mCherry-tagged constructs, and bottom bands are endogenous KRIT1. Recombinant Rap1a was detected by Ponceau S stain to verify equal loading. Representative blots and densitometry quantification are from n=3 independent experiments. Data shown are mean normalized band densities +/- SEM, normalized to WT KRIT1+Rap1-GTPγS. *$p<0.01$ by Tukey's post-hoc testing vs. WT+GTPγS. $p=0.0106$ by two-way ANOVA.

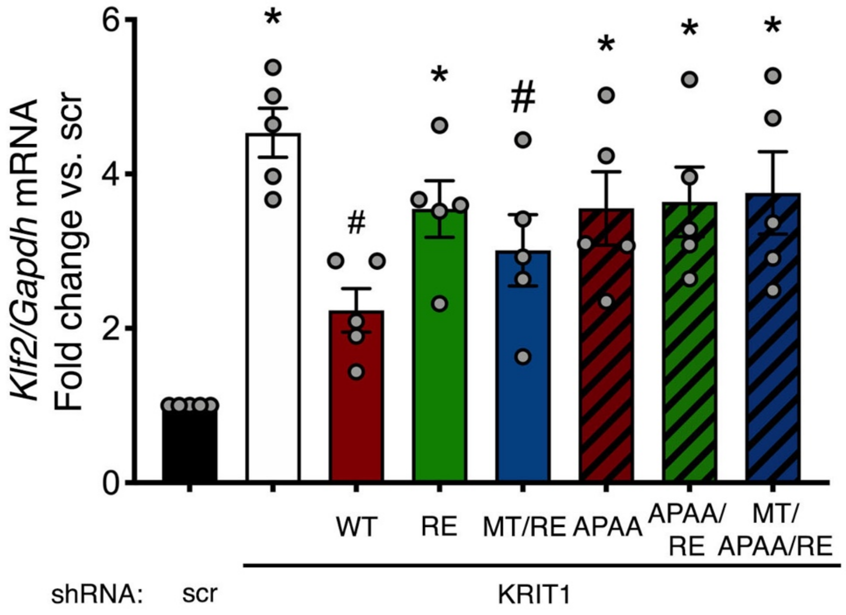

Fig. S5. *Klf2* mRNA levels are similarly rescued by KRIT1 mutant constructs. Klf2 mRNA levels were measured by semiquantitative reverse transcription PCR (RT-qPCR) in HPAEC depleted of endogenous KRIT1. Data shown are mean Klf2 mRNA levels +/- SEM, relative to *Gapdh* and normalized to scramble shRNA alone (scr), from n=5 independent experiments. *p<0.05 by Tukey post-hoc testing vs. scramble shRNA alone. #p<0.05 by Tukey post-hoc testing vs. shKRIT1 alone. p=0.0011 by one-way ANOVA.

Journal of Cell Science • Supplementary information

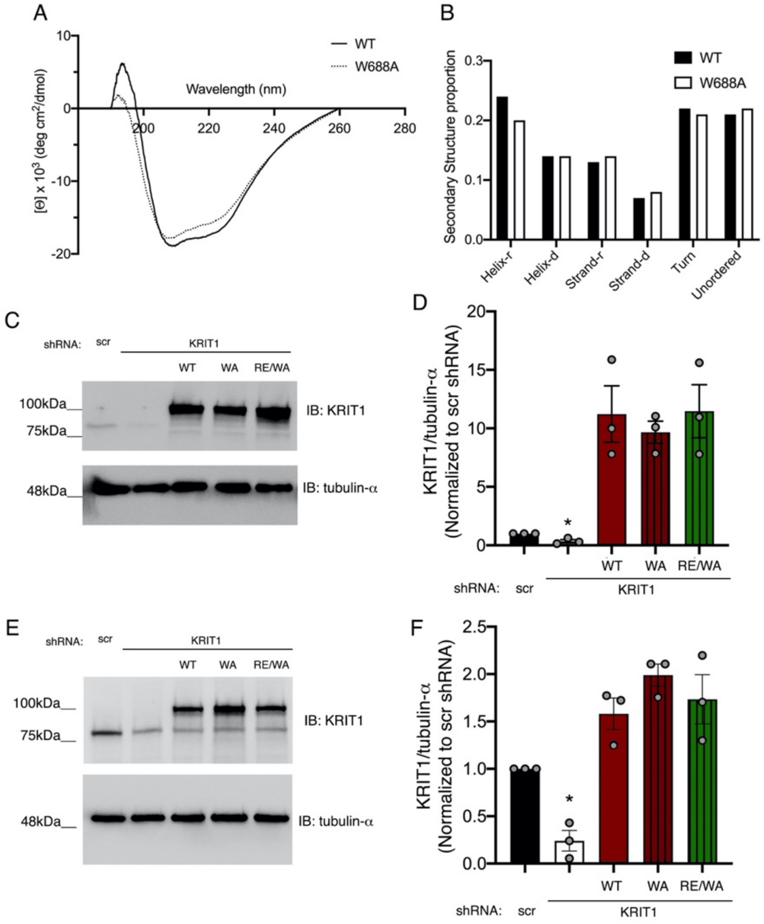

Fig S6. Circular dichroism and expression levels of KRIT1-W688A. (A) Circular dichroism spectra for WT or W688A KRIT1 FERM domain were obtained scanning from 260 to 190 nm. **(B)** Spectra in panel (A) were analyzed with Dichroweb using the CDSSTR program with reference set 4. Analysis revealed a slightly reduction in regular helical content, as defined by the program. **(C)** Overexpression of mCherry-tagged KRIT1 constructs in HPAEC after depletion of endogenous KRIT1. High adenoviral transduction yielded ~10x expression of exogenous KRIT1 relative to scramble (endogenous) control. Top KRIT1 bands correspond to mCherry-tagged constructs, and bottom bands are endogenous KRIT1. mCherry-KRIT1 band density was calculated relative to tubulin-α loading control and normalized to scramble shRNA. Representative Western blots and quantification are from n=3 independent experiments. Data shown are mean normalized band densities +/- SEM. *p<0.05 by Tukey's post-hoc testing vs. scramble shRNA alone. p=0.0184 by one-way ANOVA. **(D)** Expression of mCherry-tagged KRIT1 constructs in HPAEC after depletion of endogenous KRIT1. Adenoviral transduction yielded ~1.5x expression of exogenous KRIT1 relative to scramble (endogenous) control. Top KRIT1 bands correspond to mCherry-tagged constructs, and bottom bands are endogenous KRIT1. mCherry-KRIT1 band density was calculated relative to tubulin-α loading control and normalized to scramble shRNA. Representative Western blots and quantification are from n=3 independent experiments. Data shown are mean normalized band densities +/- SEM. *p<0.05 by Tukey's post-hoc testing vs. scramble shRNA alone. p<0.0001 by one-way ANOVA.

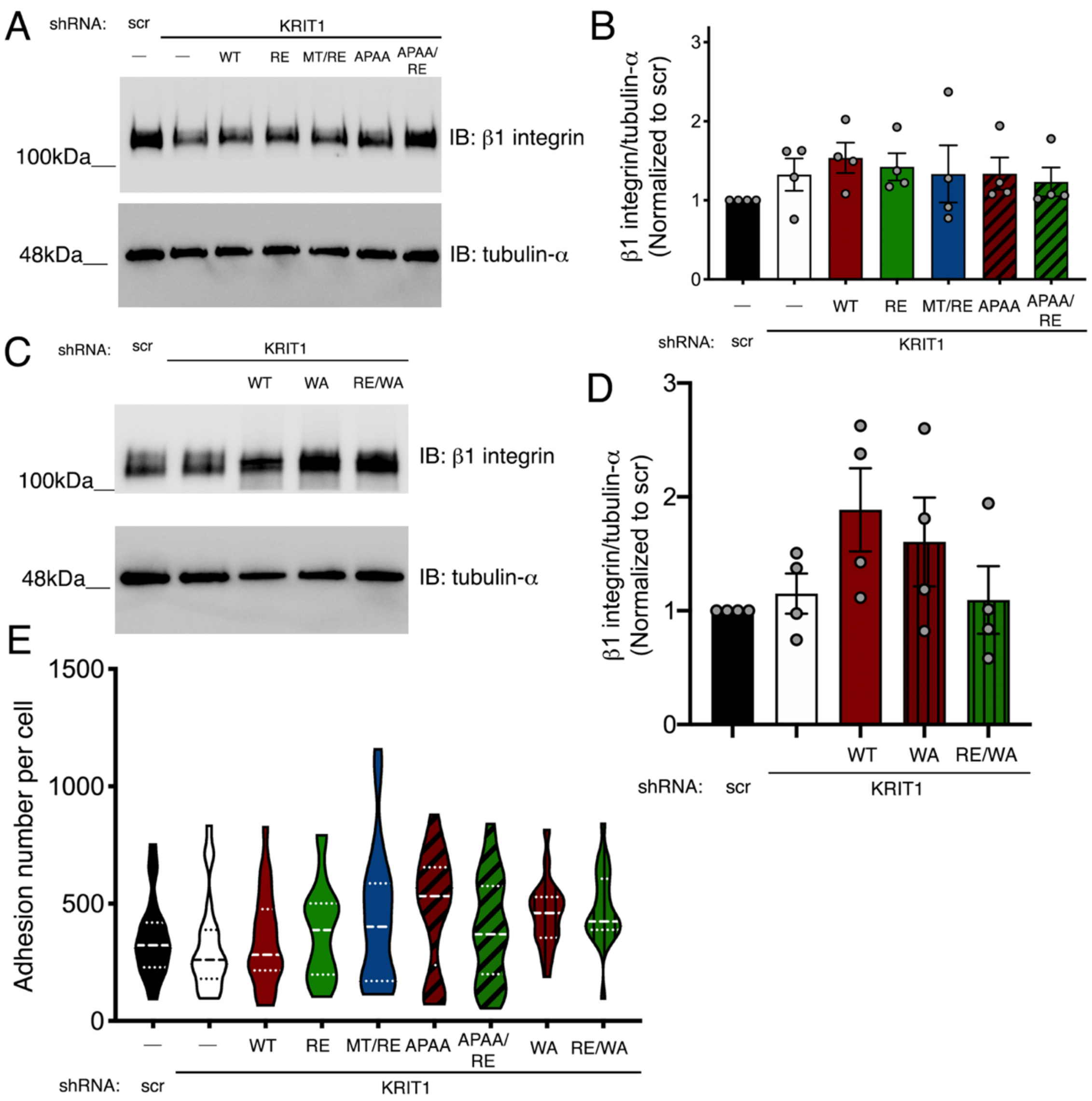

Fig. S7. Total β1 integrin expression and adhesion number are unchanged between all conditions. (A) (A-D) Expression of β1 integrin is not significantly changed in any condition. Representative Western blot of β1 integrin **(A, C)** and quantification **(B, D)** show no significant change in β1 integrin expression relative to tubulin-α (loading control) and normalized to scramble shRNA alone. Data shown in **(B)** and **(D)** are mean normalized band densities +/- SEM from n=3 independent experiments. **(E)** There was no significant change in the total number of β1 integrin-containing adhesions after staining with activation-sensitive β1 integrin antibody (HUTS4). Adhesion characteristics were calculated using Imaris image analysis software. Data are from 20-25 fields per construct across n=5 independent experiments. No significant difference by one-way ANOVA with Bonferroni's post-hoc testing. p=0.1548 by one-way ANOVA. Violin plot in **(E)** represent minimum to maximum values, with quartiles indicated by dotted lines, and median indicated by a dashed line. Data shown are from n=20-25 cells from 5 independent experiments.

Journal of Cell Science • Supplementary information
