# Supplementary material for: Contribution of protein–protein interactions to the endothelial-barrier-stabilizing function of KRIT1
Source: J Cell Sci. 2022 Jan 25;135(2):jcs258816. doi: 10.1242/jcs.258816 (PMC8917353; doi:10.1242/jcs.258816)
Supplement: Reviewer comments [file joces-135-258816_review_history.pdf]

Contribution of protein-protein interactions to the endothelial-barrier-stabilizing function of KRIT1

Harsha Swamy and Angela J. Glading
DOI: 10.1242/jcs.258816

Editor: Arnoud Sonnenberg

Review timeline

Original submission:	20 April 2021
Editorial decision:	24 May 2021
First revision received:	16 November 2021
Editorial decision:	6 December 2021
Second revision received:	7 December 2021
Accepted:	8 December 2021

Original submission

First decision letter

MS ID#: JOCES/2021/258816

MS TITLE: Contribution of protein-protein interactions to the endothelial barrier-stabilizing function of the scaffold protein KRIT1

AUTHORS: Harsha Swamy and Angela J Glading
ARTICLE TYPE: Research Article

We have now reached a decision on the above manuscript.

To see the reviewers' reports and a copy of this decision letter, please go to: https://submit-jcs.biologists.org and click on the 'Manuscripts with Decisions' queue in the Author Area. (Corresponding author only has access to reviews.)

As you will see, the reviewers consider your observations of interest but indicate that more data is needed to support your conclusion about an intramolecular of KRIT1 before they can support publication of your study. Furthermore, the reviewers call for clarity on the role of integrin activation in KRIT1-mediated junctional stabilization. For more specific details, I refer you to the comments of the reveiwers. If you think that you can deal satisfactorily with the criticisms on revision, I would be pleased to see a revised manuscript. We would then return it to the reviewers.

We are aware that you may be experiencing disruption to the normal running of your lab that makes experimental revisions challenging. If it would be helpful, we encourage you to contact us to discuss your revision in greater detail. Please send us a point-by-point response indicating where you are able to address concerns raised (either experimentally or by changes to the text) and where you will not be able to do so within the normal timeframe of a revision. We will then provide further guidance. Please also note that we are happy to extend revision timeframes as necessary.

Please ensure that you clearly highlight all changes made in the revised manuscript. Please avoid using 'Tracked changes' in Word files as these are lost in PDF conversion.

© 2021. Published by The Company of Biologists under the terms of the Creative Commons Attribution License (https://creativecommons.org/licenses/by/4.0/).

I should be grateful if you would also provide a point-by-point response detailing how you have dealt with the points raised by the reviewers in the 'Response to Reviewers' box. Please attend to all of the reviewers' comments. If you do not agree with any of their criticisms or suggestions please explain clearly why this is so.

Reviewer 1

Advance summary and potential significance to field

This manuscript by Swamy and Glading explores the mechanisms of regulation of KRIT1 function on endothelial barrier integrity. Whereas many studies have allowed to know much about the impact of KRIT1 loss on downstream endothelial signaling and behaviors, the question of how KRIT1 protein and functions are regulated still needs to be addressed. This is of high relevance to understand better the CCM disease with potential therapeutic implications and to understand the biological mechanisms that maintain the endothelial quiescence. Towards this goal, the study addresses the role of membrane targeting and of Rap1 or ICAP-1 binding onto the regulation by KRIT1 of cell-cell junction formation and permeability barrier. Moreover, it indirectly addresses the role of the intramolecular interaction of KRIT1 N- and C- terminal domains on these functions, an important point that has not been examined up to now. Their new findings suggest that Rap1 and ICAP-1 binding could control the opening of KRIT1 protein for it to exert its function on cell-cell and cell-ECM adhesion sites via an action on b1 integrin activation. Whereas there is still some way to go before ascertaining this regulatory mechanism, these results are interesting and stimulating. They open the field to new ideas and models.

Comments for the author

I suggest that several major points should be addressed.
1- To test whether the opening of KRIT1 allows its direct interaction with b1 integrin, a version of the APAA mutant deleted of its C-terminal PTB domain
(by introducing a stop codon) (Fig1A) could be studied to see whether deleting the PTB domain blocks the rescuing effect of the APAA mutant on b1 integrin activity. Indeed, a direct interaction of KRIT PTB domain with one of the 2 NPXY motifs of b1 cytoplasmic tail is not irrealistic. The authors thereby would reinforce their assumption that KRIT1 controls b1 integrin activity independently of ICAP-1.
2- Following the same idea, TIRF analysis should be performed to see whether the APAA mutants co-localize with b1 integrin.
3- Actin and pML stainings could be presented in figure 5A and 6B. This would better document the rescuing effect of the APAA mutant on cell-cell junction through its effect on the production of b1 integrin-anchored transversal actin stress fibers vs cortical actin.
4- Each figure showing immunostainings should include a photo of the shKRIT1 condition. This would facilitate the interpretation of the results.

Minor points:
1- Fig 6C and 6D legends: the number of cell analyzed and SEM are missing.
2- Fig 2A: the incomplete restoration of bcatenin staining is not obvious on this photo.
3- Lines 82 and 83: two references are missing.
4- Line 107: Beraud et al. have shown that KRIT1 is able to bind to PIP2 by itself even though they also showed that RAP1 increased this binding. So modify a bit the sentence accordingly.
5- Line 127: A reference is missing.
6- Line 358-360: The authors can also mention that KRIT-APAA does not stabilize ICAP-1 protein that is therefore degraded when they are both co-
expressed in CHO cells (Faurobert et al. JCB 2013).
Reviewed by Eva Faurobert

© 2021. Published by The Company of Biologists under the terms of the Creative Commons Attribution License (https://creativecommons.org/licenses/by/4.0/).

Reviewer 2

Advance summary and potential significance to field

This is a systematic analysis of the ability of KRIT1 mutants with altered membrane localization, ICAP1a binding, and Rap1 binding, to influence endothelial barrier formation. The results are of value as they are likely to promote a reevaluation of prior models. However, as the results are largely negative (telling us what is not involved rather than what is) and correlative, more care is needed in their explanation and interpretation. The authors promote the idea of an inhibitory intermolecular interaction and more support for this would greatly enhance the manuscript. Likewise, they postulate that altered integrin activation lies upstream of barrier defects but their data do not allow them to distinguish whether integrins are upstream of junctions or vice vera or whether integrins and junctions are on parallel pathways downstream of KRIT1. This should be addressed experimentally or if not then conclusions need to be modified accordingly.

Comments for the author

The authors have generated KRIT1 mutants containing a mutation in the Rap1-binding site that reduces (but does not fully block) binding, and a KRIT1 containing a C-terminal polybasic and CAAX motif that supports membrane targeting. Figure 1 and Fig S1 validate these constructs although the rationale for using a modestly knocked down cells for the localization studies (Fig 1D) was not well explained. As the constructs used are over-expressed what is the relevance of the ~65% knockdown of endogenous protein?
The studies rely heavily on rescue of knockdown phenotypes but Fig 2 does not show the impact of KRIT1 knockdown on b-catenin targeting. This is shown in supplementary data but it would be more convincing shown together with the rescue experiments in the same experiment.
Are the % perimeter staining and % continuous staining the best readouts for the phenotypes being observed? It looks to me like the major differences between the conditions shown relates to the thickness or ‘fuzziness’ of the catenin stain. Is this true if more cells are observed? What might this represent? Using this measure, the b-catenin pattern for RE and MT/RE are not so similar and the MT/RE is closer to WT.
Results from Fig 1-3 show that junctions and permeability are impaired by loss of KRIT1 and that this can be rescued by a tagged WT construct but not constructs with impaired Rap1 binding, even those containing a C-terminal membrane-targeting motif. Thus, despite including a relatively large amount of work, while providing further support for the already well-accepted idea that Rap1 binding is important this part of the study offers little new. It may be that Rap1 binding provides more than membrane localization but it could equally be that the tag localizes KRIT1 to a different region of the membrane (this was not carefully examined) or that adding the tag impairs other functions of KRIT1. Can WT KRIT1 (without the R/E mutation) containing the MT tag rescue barrier function?
The ICAP1 binding data in Fig 4 are confounded by problems in expressing ICAP1 in cells lacking an ICAP1-binding KRIT1. The quantitation shown does not seem to account for the lack of ICAP1 in the APAA conditions. The importance of ICAP1-KRIT1 interactions in stabilization of protein expression has precedent but makes assessment of binding more difficult and this needs to be acknowledged and the quantification methods adjusted to account for this.
The data on KLF2 in Fig S5 are glossed over in the results section - these data show that WT KRIT1 only partially rescues the KLF activation phenotype, and that the mutant constructs (with the possible exception of MT/RE are less effective at rescuing. If I read the results correctly, only WT and MT/RE showed significantly reduced KLF2 levels compared to shKRIT1 - i.e., the other constructs were unable to significantly rescue the KLF2 phenotype despite apparently binding CCM2 at normal levels.
I believe the most significant findings in the manuscript are that barrier phenotypes can be rescued with an APAA mutant (defective in ICAP1 binding and where a potential intramolecular interaction is also disrupted) and most notably that this APAA mutation restores the ability of the Rap1-binding mutant to rescue. Thus junctional phenotypes can be rescued with KRIT1 constructs that do not bind ICAP1 or Rap1 and which fail to localize at membranes or junctions. The authors interpret their findings with APAA in the context of an intermolecular interaction and additional evidence of the important of this would greatly strengthen the manuscript. They reference unpublished data that might help with this. They might also test other methods of breaking the association -

© 2021. Published by The Company of Biologists under the terms of the Creative Commons Attribution License (https://creativecommons.org/licenses/by/4.0/).

mutations in the PTB domain? If this also restored the ability of the R/E mutant to rescue barriers it would be supportive of their model.
If 'opening' of KRIT1 is important this is presumably due to binding of other proteins but they have excluded essential roles for two known partners ICAP1 and Rap1 – what about CCM2? If mutations are introduced to disrupt CCM2 binding what happens to barrier function?
The results section finishes with a correlation between changes in the area and morphology of integrin adhesions and changes in junctional/barrier function. These are interesting correlations but do not provide mechanistic information and care is needed with statements like "As the presence of higher levels of b1 integrin activation and a more centralized localization of integrin-containing adhesions correlates with the loss of stabilization of the endothelial barrier, these findings suggest KRIT1 is able to stabilize endothelial AJ and barrier function via indirect inhibition of b1 integrin." As the authors point out the results are correlative so there seems no more reason to think the integrin phenotype causes the junctional phenotype than the junctional phenotype causes the integrin phenotype, or that both occur downstream of a common KRIT1 effect.

Additional points:
Use of bar charts with SEM bars is now generally discouraged as it masks some of the variance of the population. Scatter plots showing all the data or including box and whiskers plots or violin plots would improve reporting of the results.
Citations are needed in the statement on lines 81-83 of the introduction "Furthermore, while it is well established that the KRIT1 N-terminus competes with b1 integrin for binding to ICAP1a, and while the KRIT1-
ICAP1a interaction has been suggested to be important for nuclear accumulation of both proteins"

<u>Reviewer 3</u>

Advance summary and potential significance to field

This manuscript discussed the domain contribution of KRIT1 in barrier regulation. This a important topic that is relevant to endothelial barrier maintenance and regulation. The authors defined two different domains that regulate KRIT1 's ability to regulate barrier function. The results are interesting. However, the molecular mechanisms for these domains to regulate epithelial barrier is not clear. Without this mechanistic understanding, the results presented are rather descriptive. This significantly decreased the reviewer's enthusiasm of this manuscript.

Comments for the author

Specific Comments:
1. Fig 2A. Can the authors also show images from control cells without KRIT1 knockdown and control cells with KRIT1 KD.
2. Fig. 2B-C. Can the authors also show staining strength of KRIT1 and b-cat, and KRIT1/b-cat ratio?
3. What about other know factors that can potentially regulate epithelial barrier function, including RhoA expression and activity, MLC phosphorylation KLF2/4 expression?
4. Fig. 4A. IP experiment showed that decreased APAA mutant got pulled down. However, it is not a fair comparison, because APAA mutant expression is much lower. Can the authors normalize the IP amount with the WCL amount?
5. Fig 4 showed that APAA associated mutants have much lower expression Fig 5A, S3, S4. shows a very different picture. why this is the case? How many times were the 293-overexpression experiment performed? For endothelial cell work, what is the fraction of cells with mCherry expression? Does this affect the interpretation of the results? Can the authors include RE and MT/RE mutant in the same experiment? Howe does APAA associated mutants affect RhoA expression and activity, MLC phosphorylation, KLF2/4 expression?
6. The biggest issue is how APAA site interacts with RE site to regulate epithelial function. What is the binding partner differences between APAA and RE mutants? Are there ways to determine how APAA and RE site synergistically or antagonistically affect cell-cell junction organization and endothelial barrier function

© 2021. Published by The Company of Biologists under the terms of the Creative Commons Attribution License (https://creativecommons.org/licenses/by/4.0/).

First revision

Author response to reviewers' comments

Reviewer #1:

We would like to thank the Reviewer for considering our findings *"interesting and stimulating,"* as well as their suggestions for improving the manuscript. Specific suggestions are addressed below:

1. *"a version of the APAA mutant deleted of its C-terminal PTB domain (by introducing a stop codon) (Fig1A) could be studied to see whether deleting the PTB domain blocks the rescuing effect of the APAA mutant on b1 integrin activity."*

We thank the reviewer for this suggestion. We spent considerable effort to address this concern, which culminated in the design of three different PTB domain mutants, dPTB (as suggested by the reviewer), a triple point mutant (FWL) and a single point mutant (W688A). As shown in Reviewer's Figure 1 the dPTB and FWL mutations strongly destabilized KRIT1, resulting in low protein expression (RF 1A), loss of ICAP1a association (RF 1B), and preventing purification of KRIT1 FERM domain containing these mutants (RF 1C). However, these properties were retained in the more conservative W688A mutant. Circular dichroism demonstrated that this mutation caused only a minor effect on protein folding (Fig. S6). Using this mutation, we now show that W688A rescues barrier function even in the absence of Rap1 binding or junctional localization (Fig. 6), though not to the extent shown by the APAA mutants.

2. *"Following the same idea, TIRF analysis should be performed to see whether the APAA mutants co- localize with b1 integrin."*

In accordance with the Reviewer's suggestion, TIRF microscopy was performed on HPAEC transduced with mCherry-KRIT1 constructs and stained for activated β1 integrin in order to assess whether there was any association with activated β1 integrin. KRIT1 WT, R452E, APAA, APAA-R452E constructs were all assessed. KRIT1 constructs failed to colocalize with activated β1 integrin in TIRF images, though we could observe colocalization with vinculin (Reviewer's Figure 2). This data supports work by Li et al in JBC 2012, which mentioned that there was no specific association of KRIT1 FERM domain with either β1 or β3 integrin cytoplasmic tail, based on in vitro assays (data not shown in publication). We have elected not to include this data in the current manuscript due to space limitations.

3. *"Actin and pML stainings could be presented in figure 5A and 6B."*

As suggested by the reviewer, we now include actin and pMLC staining in a new figure (Figure 8).

4. *"Each figure showing immunostainings should include a photo of the shKRIT1 condition."*

As recommended, this data is now included in figures 2, 5, 6, 7, and 8.

5. *"Fig 6C and 6D legends: the number of cell analyzed and SEM are missing."*

We thank the Reviewer for this observation, and have added the number of cells analyzed and indicated the data shown are mean+/-SEM for Figure 6 C and D (new figure 7)

6. *"Fig 2A: the incomplete restoration of b-catenin staining is not obvious on this photo."*

We agree with the reviewer that this change is subtle. We have selected another image in which it is hopefully more obvious.

7. *"Lines 82 and 83: two references are missing."*

We thank the Reviewer for noting this oversight on our part. The two citations have been added accordingly, now in lines 73 and 74.

8. *"Line 107: Beraud et al. have shown that KRIT1 is able to bind to PIP2 by itself even*

© 2021. Published by The Company of Biologists under the terms of the Creative Commons Attribution License (https://creativecommons.org/licenses/by/4.0/).

though they also showed that RAP1 increased this binding. So modify a bit the sentence accordingly."

We thank the Reviewer for this valuable point. The sentence has been edited accordingly, now in lines 100-102

9. *"Line 127: A reference is missing."*

We thank the Reviewer for noting this oversight on our part. The citation has been added accordingly, now in line 121.

10. "*Line 358-360: The authors can also mention that KRIT-APAA does not stabilize ICAP-1 protein that is therefore degraded when they are both co-expressed in CHO cells (Faurobert et al. JCB 2013*)."

We thank the Reviewer for this observation and have added this point and the relevant citation accordingly, now in lines 430-431.

Reviewer #2:

We would like to thank the Reviewer for their valuable suggestions for revising the manuscript and their suggestions for additional work that will strengthen findings initially presented. Specific suggestions are addressed below:

1. "*As the constructs used are over-expressed what is the relevance of the ~65% knockdown of endogenous protein?*"

Localization of our mCherry-KRIT1 constructs was assessed in endothelial cells with endogenous KRIT1 knockdown in order to limit potential artifacts due to the presence of endogenous KRIT1. For instance, endogenous KRIT1 could compete with our mutant constructs, which could affect either localization or function. While we agree that a more efficient knockdown would be optimal, we believe that it is important to limit potential confounding effects as much as possible.

2. "*Fig 2 does not show the impact of KRIT1 knockdown on b-catenin targeting. This is shown in supplementary data but it would be more convincing shown together with the rescue experiments in the same experiment.*"

As indicated in the response to Reviewer 1 (Response 4, above), we have added the shRNA control images all immunofluorescence figures.

3. *"Are the % perimeter staining and % continuous staining the best readouts for the phenotypes being observed?"*

We appreciate the Reviewer's careful consideration of our results. We felt that continuity and coverage of staining at the cell perimeter are appropriate as loss of continuity or coverage would suggest a disruption of the cell-cell contacts. Additionally, loss of continuity and coverage would indicate the formation of large gaps through which material may move between the apical and basal sides of the cell monolayer, thus resulting in dysregulated barrier function. However, as previous studies have also assessed junctional thickness as a measure of junctional stability, we have now included this type of analysis as well in Figures 2D and 5D. As noted by the Reviewer, our original analysis showed that the MT/RE construct partially rescued junctional staining continuity and coverage. The new analysis of junctional thickness indicates that the MT/RE construct does not rescue junctional thickness, which helps to explain why this mutant does not rescue barrier function. We thank the Reviewer for this important observation and believe that addition of this analysis augments our conclusions.

4. *" Can WT KRIT1 (without the R/E mutation) containing the MT tag rescue barrier function?"*

© 2021. Published by The Company of Biologists under the terms of the Creative Commons Attribution License (https://creativecommons.org/licenses/by/4.0/).

We thank the Reviewer for their critical analysis of our data regarding our novel membrane-targeted constructs. As we see that the membrane-targeted MT-APAA-R452E construct both localizes at cell-cell contacts while also rescuing barrier function, we feel that it is unlikely that addition of the C-terminal lipid modification negatively affects KRIT1 function (Figure 5). Additionally, we note that both MT-R452E and MT-AA-R452E are present with ß-catenin in individual confocal slices, as is the case with WT KRIT1, suggestion proper localization of these constructs. We have addressed these points in the Discussion (lines 353-357).

5. *"The ICAP1 binding data in Fig 4…does not seem to account for the lack of ICAP1 in the APAA conditions. … this needs to be acknowledged and the quantification methods adjusted to account for this."*

We appreciate the Reviewer's through assessment of our data. In accordance with their suggestion, we have modified our analysis of the KRIT1-ICAP1α co-immunoprecipitation experiment to account for the perturbed expression of ICAP1α in conditions where KRIT1-APAA constructs are co-expressed. We normalized the amount of ICAP1α co-immunoprecipitated with mCherry-KRIT1 to the expression of total ICAP1α in the whole cell lysate (WCL). This was then normalized to the amount of mCherry-KRIT1 that was immunoprecipitated ((ICAP1α IP/ICAP1α WCL)/mCherry-KRIT1 IP). The modified quantification is shown in Figure 4B. Based on this analysis, KRIT1-APAA constructs still interact with significantly less ICAP1α.

6. *"The data on KLF2 in Fig S5 are glossed over in the results section – these data show that WT KRIT1 only partially rescues the KLF activation phenotype, and that the mutant constructs (with the possible exception of MT/RE are less effective at rescuing. If I read the results correctly, only WT and MT/RE showed significantly reduced KLF2 levels compared to shKRIT1 – i.e., the other constructs were unable to significantly rescue the KLF2 phenotype despite apparently binding CCM2 at normal levels.*

In accordance with the Reviewer's comments, we have added further description and interpretation of the KLF2 expression data in lines 218-225 in the Results, and lines 469-478 in the Discussion. As the reviewer correctly states, most constructs did not have a significant effect on KLF2 mRNA levels which we believe indicates that reversing the increase in KLF2 mRNA is not an important contributor to rescue of barrier function, though it may play a role in preventing CCM lesion formation.

7. *"They might also test other methods of breaking the association – mutations in the PTB domain? If this also restored the ability of the R/E mutant to rescue barriers it would be supportive of their model."*

Thank you for this suggestion. Please see response #1 to Reviewer 1 where we detail our new findings.

8. *"…what about CCM2? If mutations are introduced to disrupt CCM2 binding what happens to barrier function?"*

We thank the Reviewer for this suggestion. Several previous studies have noted that disruption of the KRIT1-CCM2 interaction results in disrupted barrier function, concomitant with formation of actin stress fibers (Lisowka et al., J. Cell Sci. 2018 and Stockton et al., J. Exp. Med. 2010). While it would be of significant interest to assess whether this may be the result of differential KRIT1 conformation, we feel that exploring this topic will require significant work and is beyond the scope of our current study. We appreciate the Reviewer's comment and look forward to exploring this further in the future.

9. *"… care is needed with statements like "As the presence of higher levels of b1 integrin activation and a more centralized localization of integrin-containing adhesions correlates with the loss of stabilization of the endothelial barrier, these findings suggest KRIT1 is able to stabilize endothelial AJ and barrier function via indirect inhibition of b1 integrin."*

Thank you for pointing out our overreach. This has been corrected (Lines 319-321).

© 2021. Published by The Company of Biologists under the terms of the Creative Commons Attribution License (https://creativecommons.org/licenses/by/4.0/).

10. “Scatter plots showing all the data or including box and whiskers plots or violin plots would improve reporting of the results.”

In accordance with their suggestions, we have changed bar graphs for experiments of 3-5 replicates to include individual points. For experiments with more than 20 individual points (i.e. immunofluorescence quantification), we have changed the graphs to violin plots. Violin plots represent minimum to maximum values, with quartiles indicated by dotted lines, and median indicated by a dashed line.

11. “Citations are needed in the statement on lines 81-83 of the introduction” The two citations have been added accordingly, now in lines 73 and 74.

Reviewer #3:

We would like to thank the Reviewer for their supportive comments and their assertion that our study is an *“important topic that is relevant to endothelial barrier maintenance and regulation.”* We also appreciate their suggestions for improving the manuscript and adding data to strengthen our findings. Specific suggestions are addressed below:

1. *“Fig 2A. Can the authors also show images from control cells without KRIT1 knockdown and control cells with KRIT1 KD”*

We thank the Reviewer for their suggestion. As indicated in Response #4 for to Reviewer 1, we have added the shRNA control images as requested.

2. *“Fig. 2B-C. Can the authors also show staining strength of KRIT1 and b-cat, and KRIT1/b-cat ratio?”*

We now show quantification of b-catenin staining in Figures 2 and 5.

3. *“What about other know factors that can potentially regulate epithelial [sic] barrier function, including RhoA expression and activity, MLC phosphorylation, KLF2/4 expression?”*

Please see response #3 to Reviewer 1. We not include data examining actin cytoskeletal structure and MLC phosphorylation as Fig 8. KLF2 mRNA expression was/is shown in Fig. S5. We did not observe increased KLF4 expression in response to KRIT1 shRNA in our system (data not shown).

4. *“Can the authors normalize the [ICAP1a] IP amount with the WCL amount?”*

Please see response #5 for Reviewer 2.

5. *“Fig 4 showed that APAA associated mutants have much lower expression, Fig 5A, S3, S4. shows a very different picture. why this is the case?”*

Data in Figure 4 was obtained using transfected HEK293 cells, whereas Figures 5, S3, and S4 were done using adenovirally transduced endothelial cells. In figure 4, only that APAA alone mutant shows a slight reduction in expression vs WT, and no difference in expression is seen in other figures. We believe the reviewer’s keen observation is unlikely to be meaningful.

6. *“The biggest issue is how APAA site interacts with RE site to regulate epithelial function. What is the binding partner differences between APAA and RE mutants? Are there ways to determine how APAA and RE site synergistically or antagonistically affect cell-cell junction organization and endothelial barrier function.”*

We appreciate the Reviewer’s consideration of how differences in binding interactions at the first NPxY motif (APAA mutation) and Rap binding site (R452E mutation) may be affecting the phenotypes we observed. In our study, this is addressed by comparing mutants containing a single mutation (APAA or RE) or a combined mutation (APAA-RE). We are currently pursuing how these mutations affect the interaction of KRIT1 with other known and unknown partners using proximity

© 2021. Published by The Company of Biologists under the terms of the Creative Commons Attribution License (https://creativecommons.org/licenses/by/4.0/).

labeling. The results of that study will be published in a future publication.

***NOTE:** We have removed unpublished data that had been provided for the referees in confidence.*

Reviewer's Fig. 1: Triple mutation or truncation of the PTB domain results in protein misfolding. (A) mCherry-KRIT1 constructs were immunoprecipitated from HEK293A lysates co-transfected with ICAP1α-Myc and blotted for KRIT1 (upper blots) and ICAP1α-Myc (lower blots). Arrowhead indicates nonspecific band present in all lanes of IP. In whole cell lysate (input) KRIT1 blot, upper bands correspond to mCherry-tagged constructs, and bottom bands are endogenous KRIT1. Blots were stripped and reprobed for Myc. Blots are representative, n=5. **(B)** Quantification of ICAP1α-Myc band density in (A), relative to respective ICAP1α-Myc expression and KRIT1 IP, normalized to WT KRIT1. Data shown are mean normalized band densities +/- SEM. *$p<0.0001$ by Tukey post-hoc testing vs. WT. $p<0.0001$ by one-way ANOVA. Triple mutation (F686A-W688A- L690A) or truncation of the PTB domain (ΔPTB) resulted in inhibited association with ICAP1α, as well as reduced ICAP1α expression in whole-cell lysate, despite the presence of the WT NPxY motif. However, W688A point mutation associated with ICAP1α and promoted its expression, similar to WT KRIT1 control. **(C)** GST-tagged KRIT1 FERM domain containing PTB mutations was purified by affinity chromatography. GST tag was removed by on-column thrombin cleavage in buffer containing 150mM NaCl, 20mM n-octyl-ß-D-glucoside, and 20mM Tris-HCl pH=7.2. GST appeared to be successfully cleaved from all constructs, with some FERM domain peptide remaining on the GSH Sepharose after cleavage. However, only WT and W688A constructs were present in the flow-through (FT) after cleavage, F686A-W688A-L690A and ΔPTB peptides remaining adhered to the GSH Sepharose. Incubation in buffer containing 500mM NaCl and/or addition of stronger non-denaturing detergents (such as Triton-X 100) were not sufficient to recover the mutated FERM domains from the beads.

***NOTE:** We have removed unpublished data that had been provided for the referees in confidence.*

Reviewer's Fig. 2: KRIT1 constructs do not co-localize with activated ß1 integrin. Total internal reflection fluorescence (TIRF) imaging was used to assess co-localization of KRIT1 with activated ß1 integrin at the basal membrane. HPAEC depleted of endogenous KRIT1 with lentiviral shKRIT1 were adenovirally transduced with mCherry-KRIT1 constructs, and immunostained for activated ß1 integrin using AlexaFluor 488-conjugated antibody (Clone HUTS4, 1:500 dilution, EMD Millipore). As a positive control for TIRF, HPAEC were immunostained for vinculin (Clone VIN-11-5, 1:50 dilution, Novus Biologicals, Littleton, CO), followed by an AlexaFluor 564-conjugated anti-mouse IgG secondary antibody, and co-immunostained for activated ß1 integrin with AlexaFluor 488-conjugated HUTS4 antibody. Samples were illuminated using a C-TIRF Quad TIRF filter set and imaged with a Photometrics Prime BSI sCMOS camera (Teledyne Photometrics, Tucson, AZ) attached to a Nikon A1R HD laser scanning confocal microscope and 60X (n.a. 1.49) oil objective (Nikon). Images were captured using NIS-Elements C software (Nikon). Nuclei were labelled with Hoechst 33258 and imaged by widefield epifluorescence. While vinculin co-localized with activated ß1 integrin in the positive control samples, KRIT1 constructs did not appear to co-localize. R452E or APAA mutations did not influence KRIT1 staining pattern. Representative TIRF images from 6-8 fields from 2 biological replicates. Scale bar=20 µm.

© 2021. Published by The Company of Biologists under the terms of the Creative Commons Attribution License (https://creativecommons.org/licenses/by/4.0/).

Second decision letter

MS ID#: JOCES/2021/258816

MS TITLE: Contribution of protein-protein interactions to the endothelial barrier-stabilizing function of KRIT1

AUTHORS: Harsha Swamy and Angela J Glading
ARTICLE TYPE: Research Article

I am happy to inform you that in principle your paper is acceptable for publication in the J. Cell Science. The reviewers found that you have satisfactorily addressed their comments and recommend publication. However, the reviewers still have a few minor issues that will need to be addressed before your paper can be formally accepted.

To see the reviewer's comments and a copy of this decision letter, please go to: http://submit-jcs.biologists.org and click on the 'Manuscripts with Decisions' queue in the Author Area. (Corresponding author only has access to reviews.)

We are aware that you may be experiencing disruption to the normal running of your lab that makes experimental revisions challenging. If it would be helpful, we encourage you to contact us to discuss your revision in greater detail. Please send us a point-by-point response indicating where you are able to address concerns raised (either experimentally or by changes to the text) and where you will not be able to do so within the normal timeframe of a revision. We will then provide further guidance. Please also note that we are happy to extend revision timeframes as necessary.

Please ensure that you clearly highlight all changes made in the revised manuscript. Please avoid using 'Tracked changes' in Word files as these are lost in PDF conversion.

I should be grateful if you would also provide a point-by-point response detailing how you have dealt with the points raised by the reviewers in the 'Response to Reviewers' box. Please attend to all of the reviewers' comments. If you do not agree with any of their criticisms or suggestions please explain clearly why this is so.

Reviewer 1

Advance summary and potential significance to field

The authors have satisfactorily answered to my points and the manuscript is now greatly improved. It should have a strong impact in the CCM field.

Comments for the author

Two minor comments:
in some figures, bcatenin is wrongly labelled (beta is replaced by a rectangle).
The last reference does not correctly appears in the list of references.

Reviewer 2

Advance summary and potential significance to field
The authors have adequately addressed my concerns in the revised manuscript and I have only one remaining concern.

Comments for the author

The authors have adequately addressed my concerns in the revised manuscript and I have only one remaining concern. In the abstract the authors state that "… and suggest that the ability of KRIT1

© 2021. Published by The Company of Biologists under the terms of the Creative Commons Attribution License (https://creativecommons.org/licenses/by/4.0/).

to limit integrin activity is an important mechanism for barrier stabilization”. The results presented for this conclusion are purely correlative and the discussion section makes this clear. Given the available data it seems premature to conclude that the impact of KRIT1 on integrin is important for the barrier function – could the integrin effect not be downstream of barrier function, or controlled in parallel? I suggest downplaying the conclusion in the abstract.

Reviewer 3

Advance summary and potential significance to field

NA

Comments for the author

The reviewer is satisfied with the revision. In multiple figures, a box shows up where a Greek letter should be displayed. This should be corrected for publication.

Second revision

Author response to reviewers' comments

Response to Reviewers

Reviewer 1 Comments for the author

“in some figures, b catenin is wrongly labelled (beta is replaced by a rectangle). “

This has been corrected.

“The last reference does not correctly appear in the list of references.”

This has been corrected.

Reviewer 2 Comments for the author

The authors have adequately addressed my concerns in the revised manuscript and I have only one remaining concern. In the abstract the authors state that “… and suggest that the ability of KRIT1 to limit integrin activity is an important mechanism for barrier stabilization”. The results presented for this conclusion are purely correlative and the discussion section makes this clear. Given the available data it seems premature to conclude that the impact of KRIT1 on integrin is important for the barrier function –I suggest downplaying the conclusion in the abstract.

The abstract text has been changed to “and suggest that the ability of KRIT1 to limit integrin activity may be involved in barrier stabilization.”

Reviewer 3 Comments for the author

In multiple figures, a box shows up where a Greek letter should be displayed. This should be corrected for publication.

This has been corrected.

© 2021. Published by The Company of Biologists under the terms of the Creative Commons Attribution License (https://creativecommons.org/licenses/by/4.0/).

Third decision letter

MS ID#: JOCES/2021/258816

MS TITLE: Contribution of protein-protein interactions to the endothelial barrier-stabilizing function of KRIT1

AUTHORS: Harsha Swamy and Angela J Glading
ARTICLE TYPE: Research Article

I am happy to tell you that your manuscript has been accepted for publication in Journal of Cell Science, pending standard ethics checks.

© 2021. Published by The Company of Biologists under the terms of the Creative Commons Attribution License (https://creativecommons.org/licenses/by/4.0/).

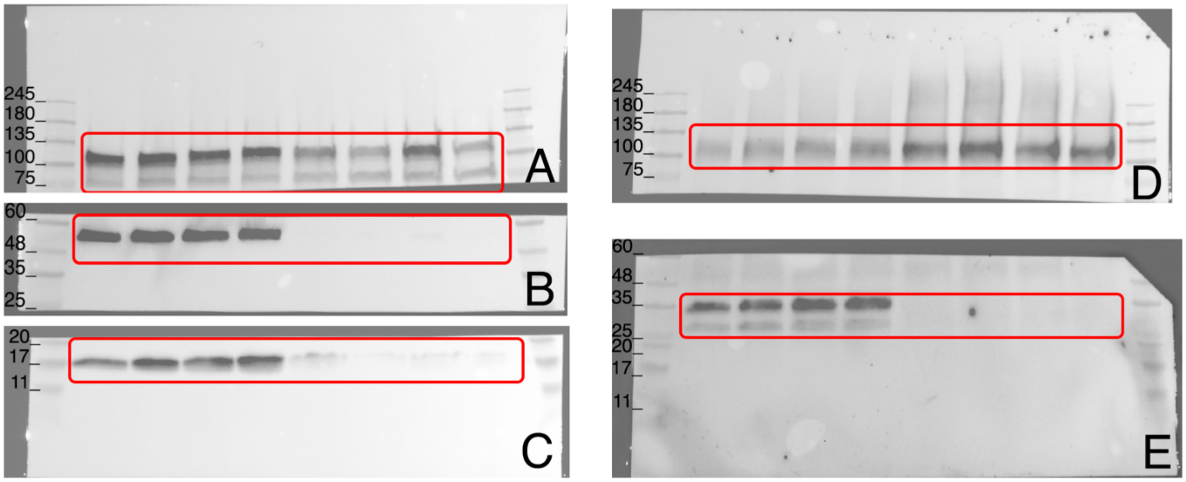

Figure 1D: Blots run in parallel. Membranes were cut and probed separately. (A) IB KRIT1. (B) IB tubulin-α. (C) IB Histone H3. (D) IB Na-K ATPase. (E) IB RhoGDI.

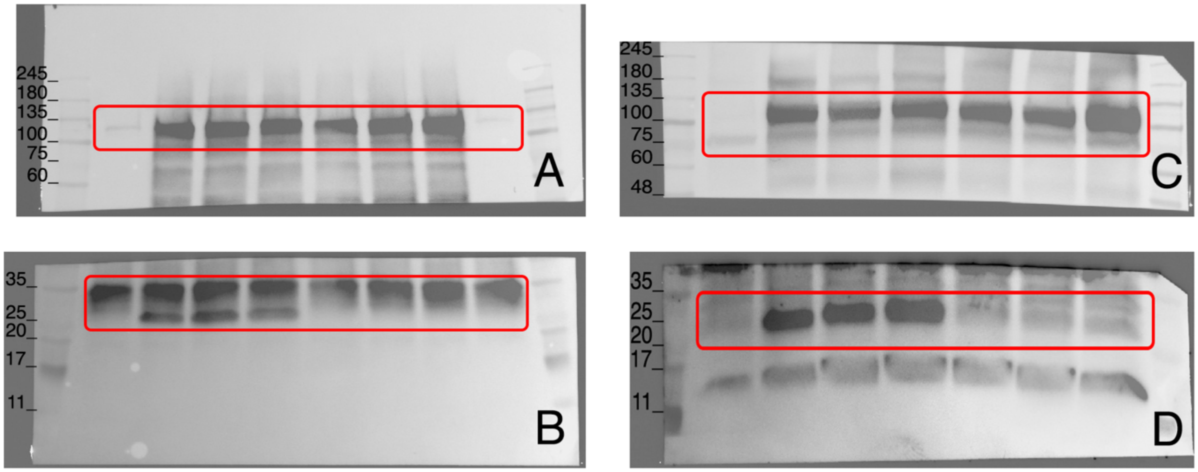

Figure 4A: IP and whole cell lysate blots run in parallel. Membranes were cut and probed separately. (A) IP mCherry, IB KRIT1. (B) IP mCherry, IB myc (ICAP1α). (C) Whole Cell lysate, IB KRIT1. (D) Whole cell lysate, IB myc (ICAP1α).

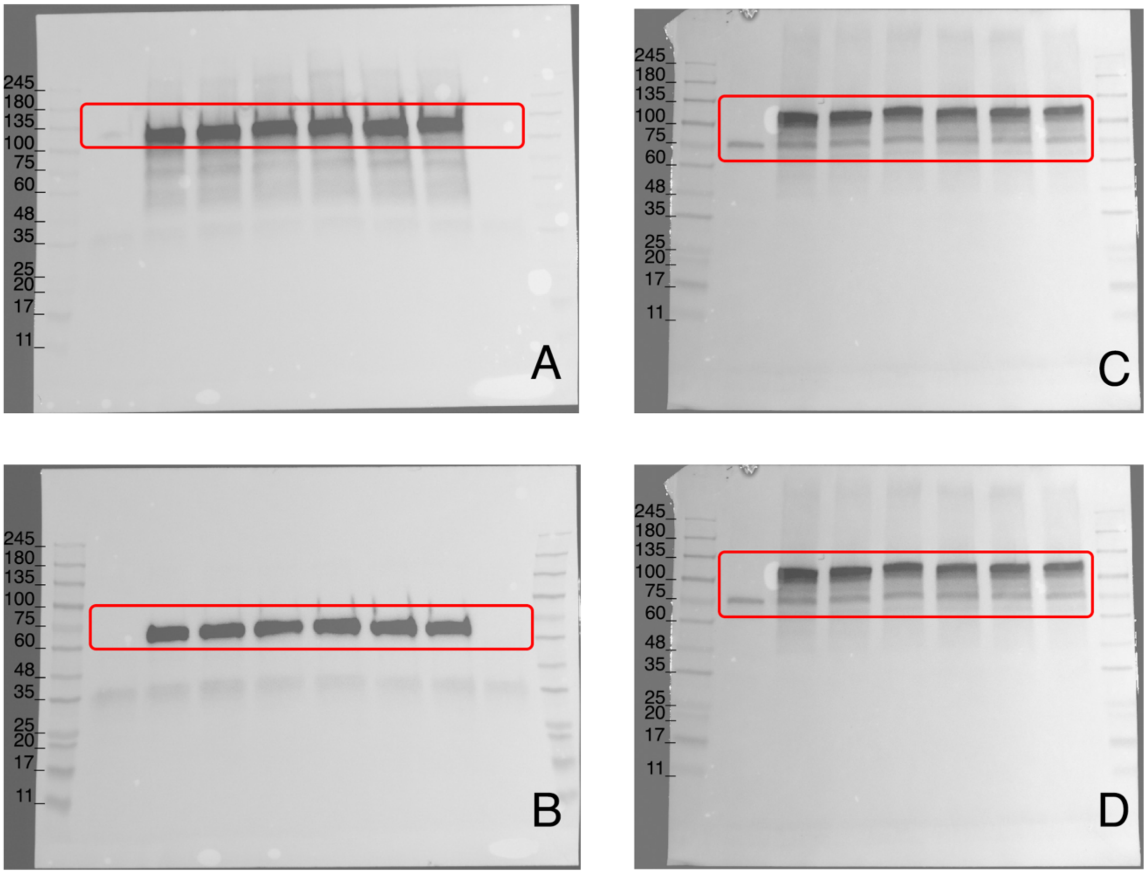

Figure 4C: IP and whole cell lysate blots run in parallel. Membranes were stripped and reprobed. (A) IP mCherry, IB KRIT1. (B) IP mCherry, IB myc (CCM2). (C) Whole Cell lysate, IB KRIT1. (D) Whole cell lysate, IB myc (CCM2).

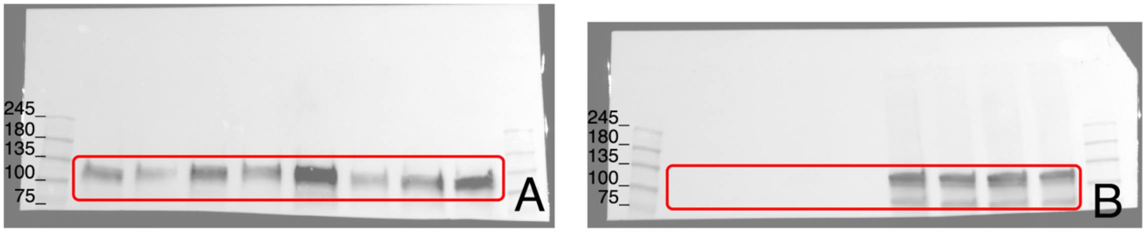

Figure S1A: Samples run on two blots in parallel. Membranes cut prior to probing together for KRIT1. (A) Rap1-GDP (lanes 2-5) and Rap1-GST (lanes 6-9) pulldown. (B) GST pulldown (lanes 2-5) and whole cell lysate control (lanes 6-9).

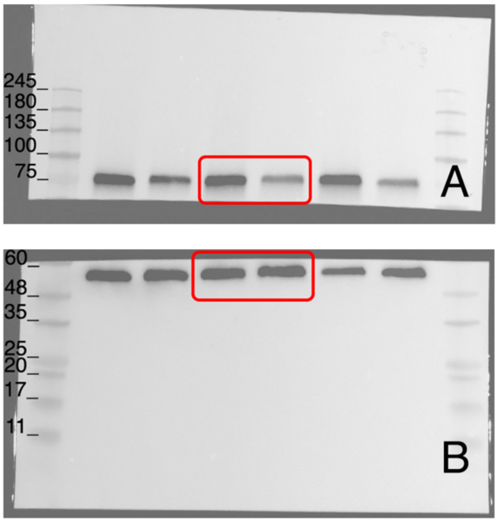

Figure S2A: Membranes cut and probed separately. (A) IB KRIT1. (B) IB tubulin-α.

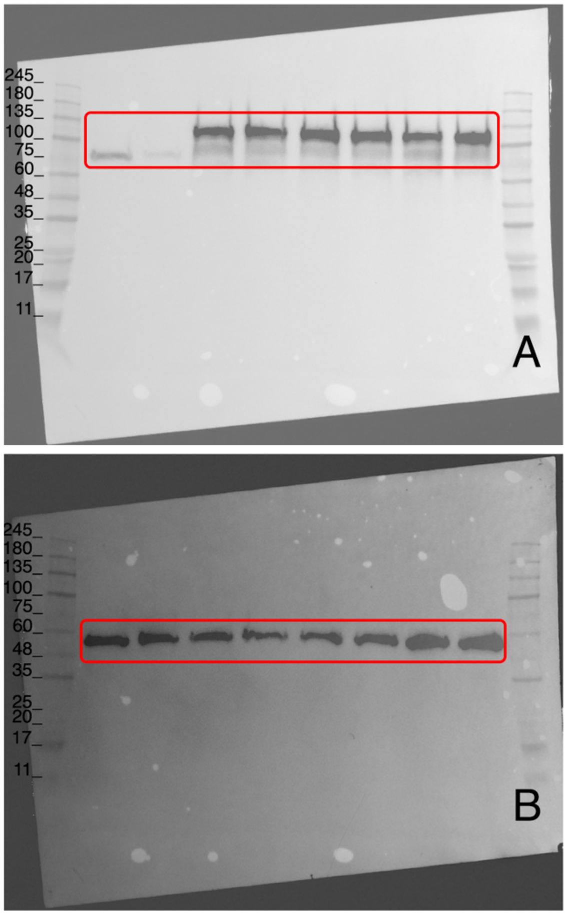

Figure S2C: Membranes stripped and reprobed. (A) IB KRIT1. (B) IB tubulin-α.

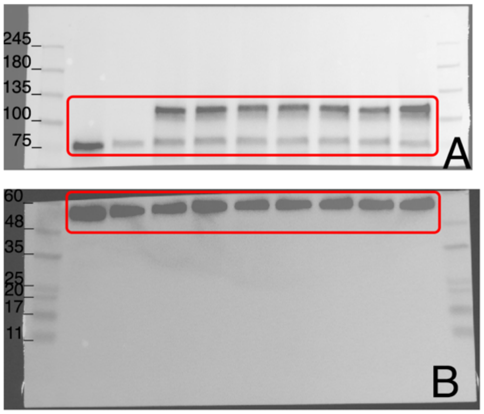

Figure S3: Membranes cut and probed separately. (A) IB KRIT1. (B) IB tubulin-α.

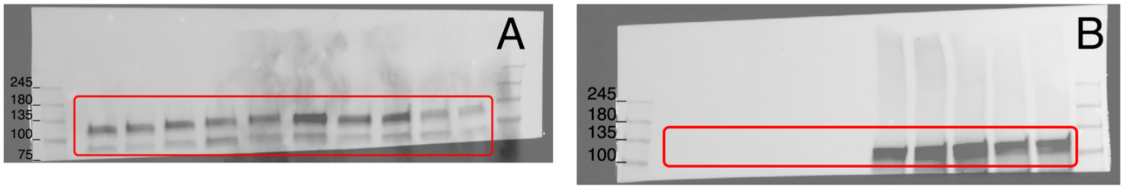

Figure S4A: Samples run on two blots in parallel. Membranes cut prior to probing together for KRIT1. (A) Rap1-GDP (lanes 2-6) and Rap1-GST (lanes 7-11) pulldown. (B) GST pulldown (lanes 2-6) and whole cell lysate control (lanes 7-11).

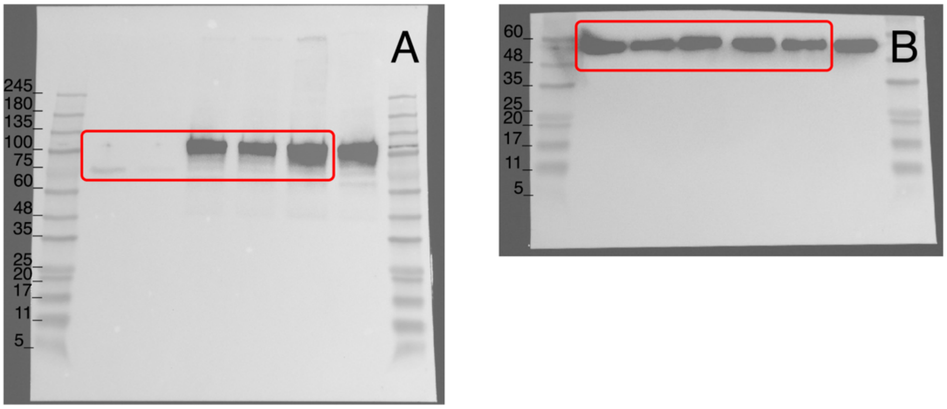

Figure 6C: (A) IB KRIT1. (B) Blot stripped, cut, and reprobed for tubulin-α (top portion used for a separate experiment).

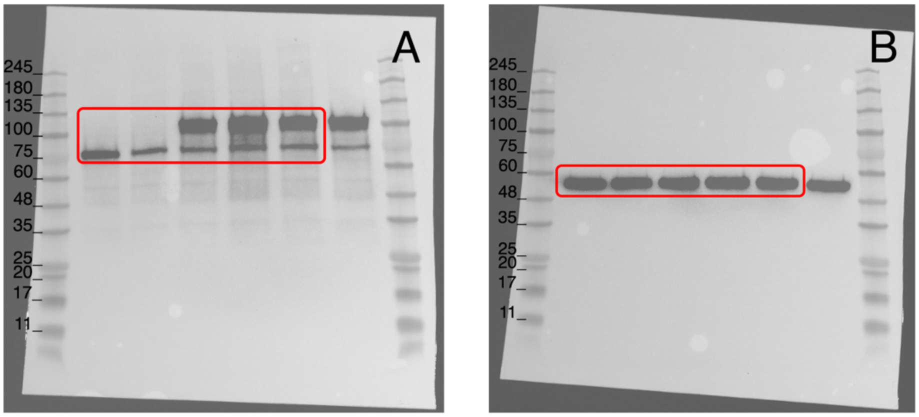

Figure 6E: Membranes stripped and reprobed. (A) IB KRIT1. (B) IB tubulin-α.

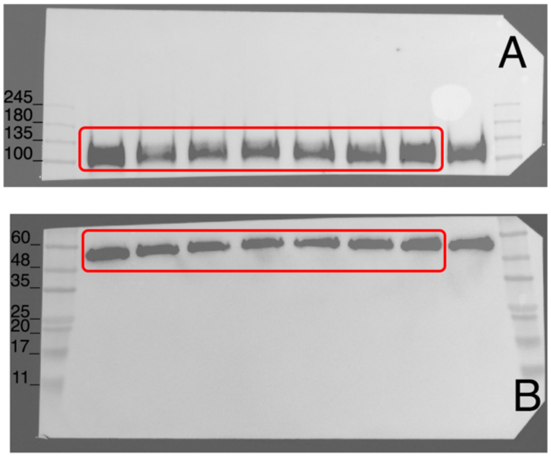

Figure 7A: Membranes cut and probed separately. (A) IB β1 integrin. (B) IB tubulin-α.

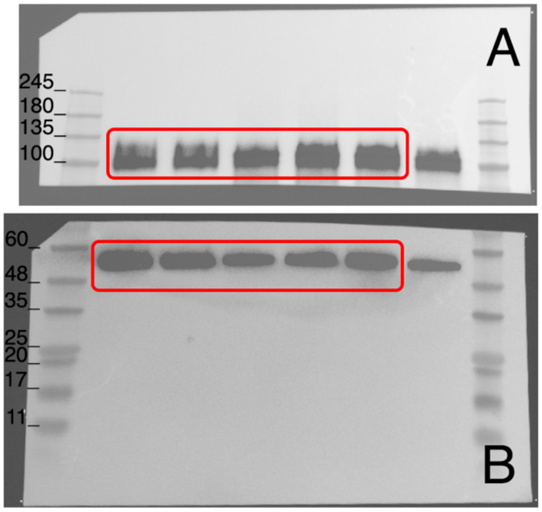

Figure 7C: Membranes cut and probed separately. (A) IB β1 integrin. (B) IB tubulin-α.
